# QCD next-to-leading-order predictions matched to parton showers for vector-like quark models

**DOI:** 10.1140/epjc/s10052-017-4686-z

**Published:** 2017-02-27

**Authors:** Benjamin Fuks, Hua-Sheng Shao

**Affiliations:** 1 Sorbonne Universités, UPMC Univ. Paris 06, UMR 7589, LPTHE, 75005 Paris, France; 2 CNRS, UMR 7589, LPTHE, 75005 Paris, France; 3 Institut Universitaire de France, 103 Boulevard Saint-Michel, 75005 Paris, France; 4 Theoretical Physics Department, CERN, 1211 Geneva 23, Switzerland

## Abstract

Vector-like quarks are featured by a wealth of beyond the Standard Model theories and are consequently an important goal of many LHC searches for new physics. Those searches, as well as most related phenomenological studies, however, rely on predictions evaluated at the leading-order accuracy in QCD and consider well-defined simplified benchmark scenarios. Adopting an effective bottom-up approach, we compute next-to-leading-order predictions for vector-like-quark pair production and single production in association with jets, with a weak or with a Higgs boson in a general new physics setup. We additionally compute vector-like-quark contributions to the production of a pair of Standard Model bosons at the same level of accuracy. For all processes under consideration, we focus both on total cross sections and on differential distributions, most these calculations being performed for the first time in our field. As a result, our work paves the way to precise extraction of experimental limits on vector-like quarks thanks to an accurate control of the shapes of the relevant observables and emphasise the extra handles that could be provided by novel vector-like-quark probes never envisaged so far.

## Introduction

The Standard Model of particle physics is a successful theory of nature, although it exhibits many conceptual issues, like the hierarchy problem or the strong *CP* problem, and practical limitations like the absence of a viable candidate for explaining the dark matter pervading our universe. As a result, it is commonly acknowledged as an effective theory stemming from a more fundamental setup that has still to be observed and confirmed experimentally. This effective description has been recently strengthened with the discovery of a Higgs boson with properties very similar to those expected from the Standard Model in 2012. The null results of all collider searches for new particles predicted by most of beyond the Standard Model theories are, however, at the same time pushing the limits on the mass of these potential new particles to higher and higher energy scales.

Among the viable options for new physics, many extensions of the Standard Model predict the existence of additional quark species that should be observable during the next runs of the Large Hadron Collider (LHC) at CERN. One of the common feature of such theories consists of the predicted vector-like nature of the additional quarks, *i.e.* their left-handed and right-handed components lie in the same representation of the electroweak symmetry group. These quarks appear for instance in models with extra space-time dimensions that exhibit an extended gauge symmetry or a new strong dynamics giving rise to massive composite states [1–5]. As a result, vector-like quark searches play an important role in the ATLAS and CMS experimental program.

Current searches, relying on signatures induced by both the vector-like quark pair-production and single-production modes, impose strong constraints on the masses of the heavy quarks that are now bounded to be above about 750–1500 GeV [6–12], this wide range reflecting the wealth of options for describing how the new states decay into a pair of Standard Model particles. Care must, however, be taken with the interpretation of these limits as they are extracted once various simplifying assumptions are accounted for the new physics signal. Most of the bounds indeed assume that the quark partners decay, with a branching ratio of 100%, into third-generation top or bottom quarks, while more general situations where decays into second or first generation quarks are possible are less explored yet. Sizeable couplings to light quarks are still allowed by indirect constraints [13–15], which could have a severe impact on electroweak vector-like quark processes at the LHC [16]. The latter, which induce vector-like quark decays, are driven by the couplings of the extra quarks to the weak and Higgs bosons. They admit a simple model-independent parameterisation [17] regardless of the representation of the new physics particles under the electroweak symmetry group, and this bottom-up approach is now adopted by the experimental collaborations. Most LHC searches for heavy quarks hence turn out to be agnostic of the ultraviolet completion of the model and can rather easily be reinterpreted in any framework, and options for combining different searches can also be considered.

Current vector-like quark searches, as well as most associated theoretical work, are, however, based on Monte Carlo simulations of the new physics signals where hard-scattering matrix-elements are evaluated at the leading-order (LO) accuracy in QCD. In addition, event samples featuring different final-state jet multiplicities are sometimes also merged in order to get a better control on the shapes of the key differential distributions. The formal precision of these calculations is nonetheless rather limited, which directly impacts the extraction of any limit on the properties of the new particle properties or the corresponding measurements in the case of a discovery. In this work, we build up a new procedure that allows us to make use of the MadGraph5_aMC@NLO framework [18] to compute total rates and differential distributions at the next-to-leading-order (NLO) accuracy in QCD for processes involving vector-like quarks. This more precisely concerns vector-like quark production on the one hand, and the production of Standard Model particles when vector-like quark diagrams contribute, regardless of the strong or electroweak nature of the Born process.

Our methodology relies on the joint use of the FeynRules [19] and NLOCT [20] packages, the latter making use of FeynArts [21], for automatically generating a UFO library [22] that contains all tree-level vertices and counterterms necessary for NLO QCD computations. This UFO library can then be further used by MadGraph5_aMC@NLO for event generation, at the LO and NLO accuracies in QCD as well as for loop-induced processes. Virtual loop contributions are numerically evaluated through the MadLoop module [23] and combined with the real-emission diagrams following the FKS subtraction method as implemented in MadFKS [24, 25], and the matching to parton showers is finally achieved according to the MC@NLO prescription [26].

In Sect. 2, we detail how we have modified the model-independent parameterisation of Ref. [17] to make it suitable for NLO calculations in QCD matched to parton showers for vector-like quark processes. We also define a series of benchmark scenarios for the phenomenological studies performed in Sect. 3. We investigate vector-like quark pair production and single production (in association with either a jet or a weak gauge or Higgs boson), as well as diboson production. We summarise our work in Sect. 4.

## A model-independent parameterisation for vector-like quark models

### Model description

Vector-like quarks appear in many extensions of the Standard Model. They are usually included as fields lying in the fundamental representation of $SU(3)_c$ and carry colour charges similar to those of their Standard Model counterparts. However, they can lie in various representations of the weak interaction symmetry group $SU(2)_L$ and be assigned different hypercharge $U(1)_Y$ quantum numbers. Focusing on phenomenologically viable minimal models that comprise a single Standard Model Higgs field $\varPhi$, only weak triplets, doublets and singlets of vector-like quarks are allowed [27]. Consequently, the particle content of the theory can solely include four species of extra quarks, which we denote by *X*, *T*, *B* and *Y*, their respective electric charges being $Q=5/3$, 2 / 3, $-1/3$ and $-4/3$.

Although vector-like and Standard Model quarks with the same electric charge mix, the mixing pattern and the resulting phenomenology can be simplified when minimality requirements are imposed. Since we consider that the Higgs sector contains a single Standard-Model-like scalar Higgs field $\varPhi$, quark mixings are solely generated by its Yukawa interactions. The mass splitting between the vector-like quarks is consequently also constrained to be small and connected to the Higgs vacuum expectation value *v*, so that the extra quarks will always directly decay into a gauge or Higgs boson and one of the Standard Model quarks [27]. A model-independent effective parameterisation apt to describe the phenomenology of this vector-like quark setup has been recently proposed [17], but it is not suitable for higher-order QCD calculations. The reason is that in the latter parameterisation, the strengths of the interactions of the vector-like and Standard Model quarks with a single Higgs boson depend on the masses of the model particles. As a result, the renormalisation of the quark masses and the one of the couplings are related, which prevents all ultraviolet divergences that arise at the NLO from cancelling. We therefore modify the modelling of Ref. [17] so that all the couplings of the vector-like quarks to a gauge or a Higgs boson are free parameters, and we have the following effective Lagrangian:1being supplemented to the Standard Model Lagrangian $\mathcal{L}_\mathrm{SM}$. The terms in the first and second lines consist of gauge-invariant kinetic and mass terms for the vector-like quark fields (taken in the mass eigenbasis) after restricting the covariant derivatives to their QCD component,

2 $$\begin{aligned} D_\mu = \partial _\mu -i g_s T_a G_\mu ^a. \end{aligned}$$

The coupling parameter $g_s$ denotes the strong coupling constant, $G_\mu$ the gluon field and *T* (and *f* for further references) the fundamental representation matrices (the structure constants) of *SU*(3). Although the electroweak pieces of the covariant derivative could have been included, they have been omitted in order to simplify our model description since they are model-dependent and are expected to yield a negligible effect with respect to their strong interaction part.

The third and fourth lines of Eq. (1) collects the effective interactions of the physical Higgs boson *h* with one Standard Model quark and its vector-like partner, generation indices being understood for clarity. As mentioned above, such interactions are yielded by the (flavour-changing) Yukawa couplings of the Higgs doublet $\varPhi$ with the up-type ($q_u$), down-type ($q_d$) and vector-like quarks that induce a mixing of the Standard Model and the new physics quark sectors. The relevant elements of the mixing matrices have been included in the strengths of the effective interactions $\hat{\kappa }$.

In the last six lines of Eq. (1), we include the weak interactions of the *Z*-boson and *W*-boson with one Standard Model quark and one vector-like quark. In our conventions, we have factorised out the weak coupling *g* which represents the overall interaction strength, and the $\kappa$ and $\tilde{\kappa }$ parameters include the relevant elements of the quark mixing matrices, as for the Higgs interactions. Moreover, the $c_{\scriptscriptstyle W}$ parameter stands for the cosine of the electroweak mixing angle.

The $\mathcal{L}_\mathrm{VLQ}$ Lagrangian above is equivalent, at the tree level, to the one of Ref. [17] once we impose

3 $$\begin{aligned} \begin{aligned} (\hat{\kappa }^{\scriptscriptstyle Q}_{\scriptscriptstyle L,R})_f&=\frac{\kappa _{\scriptscriptstyle Q}m_{\scriptscriptstyle Q}}{v} \sqrt{\frac{\zeta _{\scriptscriptstyle L,R}^f\ \xi ^{\scriptscriptstyle Q}_{\scriptscriptstyle H}}{\varGamma ^{\scriptscriptstyle Q}_{\scriptscriptstyle H}}}, \\ (\tilde{\kappa }^{\scriptscriptstyle Q}_{\scriptscriptstyle L,R})_f&=\kappa _{\scriptscriptstyle Q} \sqrt{\frac{\zeta _{\scriptscriptstyle L,R}^f\ \xi ^{\scriptscriptstyle Q}_{\scriptscriptstyle Z}}{\varGamma ^{\scriptscriptstyle Q}_{\scriptscriptstyle Z}}},\\ (\kappa ^{\scriptscriptstyle Q}_{\scriptscriptstyle L,R})_f&=\kappa _{\scriptscriptstyle Q} \sqrt{\frac{\zeta _{\scriptscriptstyle L,R}^f\ \xi ^{\scriptscriptstyle Q}_{\scriptscriptstyle W}}{\varGamma ^{\scriptscriptstyle Q}_{\scriptscriptstyle W}}}. \end{aligned} \end{aligned}$$

In this notation, *f* stands for a generation index and $\varGamma ^{\scriptscriptstyle Q}_{\scriptscriptstyle X}$ denotes the kinematic factor of the partial decay width of the extra quark *Q* into a final state containing an *X* boson. The $\kappa _{\scriptscriptstyle Q}$ parameter encodes the magnitude of the coupling of the extra quark *Q* to the different electroweak bosons, while the $\zeta$ parameters refer to the mixing of the vector-like quarks with the Standard Model quarks,

4 $$\begin{aligned} \zeta _{\scriptscriptstyle L,R}^f = \frac{|(V_{\scriptscriptstyle L,R})_{4f}|^2}{\sum _{i=1}^3 |(V_{\scriptscriptstyle L,R})_{4j}|^2} \qquad \text {with}\quad \sum _{i=1}^3 \zeta _{\scriptscriptstyle L,R}^i = 1. \end{aligned}$$

In this expression, we have represented the left-handed and right-handed $4 \times 4$ mixing matrices between the new quarks and the three Standard Model quarks by $V_{\scriptscriptstyle L,R}$. Finally, the $\xi$ parameters of Eq. (3) determine the relative importance of the various decay modes of the vector-like quarks, their sum being equal to one.

The calculation of differential and total cross sections for LHC processes at the NLO accuracy in QCD necessitates to evaluate, on the one hand, real-emission squared amplitudes and on the other hand, interferences of tree-level with virtual one-loop diagrams. The ultraviolet divergences that arise in the latter case are absorbed through the renormalisation of the fields and parameters appearing in $\mathcal{L}_\mathrm{VLQ}$. This is achieved by replacing all fermionic and non-fermionic bare fields $\varPsi$ and $\varPhi$ and bare parameters *y* by the corresponding renormalised quantities,

5 $$\begin{aligned} \begin{aligned}&\varPhi \rightarrow \left[ 1 + \frac{1}{2} \delta Z_\varPhi \right] \varPhi ,\\&\varPsi \rightarrow \left[ 1+\frac{1}{2} \delta Z^L_\varPsi P_L + \frac{1}{2} \delta Z^R_\varPsi P_R \right] \varPsi , \\&y \rightarrow y + \delta y, \end{aligned} \end{aligned}$$

where we truncate the renormalisation constants $\delta Z$ and $\delta y$ at the first order in the strong coupling $\alpha _s=g_s^2/(4 \pi )$. While the wave-function renormalisation constants of the Standard Model quarks are not modified by the presence of the vector-like quarks, the one of the gluon field is given, when the on-shell renormalisation scheme is adopted and when we include $n_f=5$ massless flavours of quarks, by

6 $$\begin{aligned} \delta Z_g= & {} -\frac{g_s^2}{24 \pi ^2} \sum _{q=t,T,B,X,Y}\left\{ - \frac{1}{3} + B_0(0,m_q^2,m_q^2) \right. \nonumber \\&\left. \phantom {\frac{g_s^2}{24 \pi ^2}}+\, 2 m_q^2 B_0'(0,m_q^2,m_q^2)\right\} , \end{aligned}$$

where the $B_{0,1}$ and $B_{0,1}'$ functions stand for standard two-point Passarino–Veltman integrals and their derivatives [28]. The left-handed and right-handed wave function renormalisation constants $\delta Z_{\scriptscriptstyle Q}^{L,R}$ and the mass renormalisation constants $\delta m_{\scriptscriptstyle Q}$ of a vector-like quark *Q* (with $Q=T$, *B*, *X*, *Y*) are similar to the top-quark ones and read

7 $$\begin{aligned} \delta Z_{\scriptscriptstyle Q}^{L,R}= & {} \frac{g_s^2 C_F}{16 \pi ^2}[ 1 + 2 B_1(m_{\scriptscriptstyle Q}^2;m_{\scriptscriptstyle Q}^2,0)\nonumber \\&+\,8 m_{\scriptscriptstyle Q}^2 B_0'(m_{\scriptscriptstyle Q}^2; m_{\scriptscriptstyle Q}^2,0) + 4 m_{\scriptscriptstyle Q}^2 B_1'(m_{\scriptscriptstyle Q}^2; m_{\scriptscriptstyle Q}^2,0)], \nonumber \\ \delta m_{\scriptscriptstyle Q}= & {} \frac{g_s^2 C_F\ m_{\scriptscriptstyle Q}}{16 \pi ^2}[ 1 - 4 B_0(m_{\scriptscriptstyle Q}^2; m_{\scriptscriptstyle Q}^2,0) \nonumber \\&-2 B_1(m_{\scriptscriptstyle Q}^2; m_{\scriptscriptstyle Q}^2,0)]. \end{aligned}$$

In these two expressions, $C_F=(n_c^2-1)/(2 n_c)$ is the quadratic Casimir invariant associated with the fundamental representation of *SU*(3), with $n_c=3$. Finally, in order to fix the renormalisation group running of $\alpha _s$ so that it originates from gluons and the $n_f=5$ active light quark flavours, we renormalise $\alpha _s$ by subtracting, from the gluon self-energy, the contributions of all massive particles evaluated at zero-momentum transfer,

8 $$\begin{aligned} \frac{\delta \alpha _s}{\alpha _s} = \frac{\alpha _s}{2\pi \bar{\epsilon }} \left[ \frac{n_f}{3} \!-\! \frac{11 n_c}{6}\right] \!+\! \frac{\alpha _s}{6\pi }\sum _{q=t,T,B,X,Y} \left[ \frac{1}{\bar{\epsilon }} \!-\! \log \frac{m_q^2}{\mu _R^2}\right] ,\nonumber \\ \end{aligned}$$

where the ultraviolet-divergent pieces are written in terms of $\frac{1}{\bar{\epsilon }}=\frac{1}{\epsilon } - \gamma _E + \log {4\pi }$ with $\gamma _E$ being the Euler–Mascheroni constant and $\epsilon$ being connected to the number of space-time dimensions $D=4-2\epsilon$. The first term in the right-hand side of Eq. (8) results from the Standard Model massless parton contributions, while the second term is connected to the massive states, namely the top quark and the four considered vector-like quark species.

In Sect. 3, we will compute predictions at the NLO accuracy in QCD for processes involving vector-like quarks. We will rely on a numerical evaluation of the loop integrals in four dimensions, which necessitates the calculation of rational terms associated with the $\epsilon$-dimensional pieces of the loop integrals. There exist two sets of such rational terms that are, respectively, connected to the loop-integral denominators ($R_1$) and numerators ($R_2$). While the former are universal, the latter are model-dependent and can be seen as a finite number of counterterm Feynman rules derived from the bare Lagrangian [29]. Starting from the $\mathcal{L}_\mathrm{VLQ}$ Lagrangian of Eq. (1), several $R_2$ counterterms with external gauge bosons are modified with respect to the Standard Model case,

9 $$\begin{aligned} R_2^{G G}= & {} \frac{i g_s^2}{96 \pi ^2}\left[ (9 p_2^2 \eta ^{\mu _1\mu _2} - 6 p_2^{\mu _1} p_2^{\mu _2})\phantom {\sum _q}\right. \nonumber \\&\left. +\, 2 \eta ^{\mu _1\mu _2}\sum _q\{p_2^2-6m_q^2\}\right] \delta _{c_1c_2}, \nonumber \\ R_2^{G G G}= & {} -\frac{g_s^3 f_{c_1c_2c_3}}{192 \pi ^2}\left( 33+\sum _q8 \right) V^{\mu _1\mu _2\mu _3},\nonumber \\ R_2^{G G G G}= & {} \frac{i g_s^4}{48 \pi ^2}\Big ( C^{(1)}_{c_1c_2c_3c_4} \eta ^{\mu _1\mu _2} \eta ^{\mu _3\mu _4} \nonumber \\&+\, C^{(2)}_{c_1c_2c_3c_4} \eta ^{\mu _1\mu _3} \eta ^{\mu _2\mu _4}+ C^{(3)}_{c_1c_2c_3c_4} \eta ^{\mu _1\mu _4} \eta ^{\mu _2\mu _3} \Big ),\nonumber \\ R_2^{WWGG}= & {} \frac{i g^2 g_s^2}{96 \pi ^2} V^{\mu _1\mu _2\mu _3\mu _4}\nonumber \\&\times \left( 3+\sum _{Q,f} \Big \{ (\kappa ^{\scriptscriptstyle Q}_{\scriptscriptstyle L})_f^2+ (\kappa ^{\scriptscriptstyle Q}_{\scriptscriptstyle R})_f^2\Big \}\right) \delta _{c_3c_4},\nonumber \\ R_2^{ZZGG}= & {} \frac{i g^2 g_s^2}{288 c_{\scriptscriptstyle W}^2 \pi ^2} V^{\mu _1\mu _2\mu _3\mu _4} \left( 8-18s_{\scriptscriptstyle W}^2 + 20 s_{\scriptscriptstyle W}^4\phantom {\sum _{Q,f}}\right. \nonumber \\&\left. +\,3 \sum _{Q,f} \Big \{ (\tilde{\kappa }^{\scriptscriptstyle Q}_{\scriptscriptstyle L})_f^2+ (\tilde{\kappa }^{\scriptscriptstyle Q}_{\scriptscriptstyle R})_f^2\Big \}\right) \delta _{c_3c_4},\nonumber \\ R_2^{GGhh}= & {} \frac{i g_s^2}{16 \pi ^2}\nonumber \\&\times \eta ^{\mu _1\mu _2} \left( -y_t^2 -2 \sum _{Q,f} \Big \{ (\hat{\kappa }^{\scriptscriptstyle Q}_{\scriptscriptstyle L})_f^2+(\hat{\kappa }^{\scriptscriptstyle Q}_{\scriptscriptstyle R})_f^2\Big \} \right) \delta _{c_3c_4},\nonumber \\ \end{aligned}$$

with

10 $$\begin{aligned}&V^{\mu _1\mu _2\mu _3}=(p_1-p_2)^{\mu _3}\eta ^{\mu _1\mu _2} + (p_3-p_1)^{\mu _2}\eta ^{\mu _3\mu _1}\nonumber \\&\quad +\, (p_2-p_3)^{\mu _1}\eta ^{\mu _2\mu _3}, \nonumber \\&V^{\mu _1\mu _2\mu _3\mu _4}=\eta ^{\mu _1\mu _2} \eta ^{\mu _3\mu _4} \!+\! \eta ^{\mu _1\mu _3} \eta ^{\mu _2\mu _4} \!+\! \eta ^{\mu _1\mu _4} \eta ^{\mu _2\mu _3}, \nonumber \\&C_{abcd}^{(1)}=\delta _{ad}\delta _{bc}+\delta _{ac}\delta _{bd}+\delta _{ab}\delta _{cd}\nonumber \\&\quad +\, 2 (\mathrm{Tr}[T_aT_cT_bT_d]+\mathrm{Tr}[T_aT_dT_bT_c])\left( 45+\sum _q11\right) \nonumber \\&\quad -\, 6 (\mathrm{Tr}[T_aT_bT_cT_d]+\mathrm{Tr}[T_aT_bT_dT_c])\left( 7+\sum _q2\right) \nonumber \\&\quad -\,6 (\mathrm{Tr}[T_aT_cT_dT_b]+ \mathrm{Tr}[T_aT_dT_cT_b])\left( 7+\sum _q2\right) , \nonumber \\&C_{abcd}^{(2)} = \delta _{ad}\delta _{bc}+\delta _{ac}\delta _{bd}+\delta _{ab}\delta _{cd} \nonumber \\&\quad -\, 6 (\mathrm{Tr}[T_aT_bT_dT_c]+ \mathrm{Tr}[T_aT_cT_dT_b])\left( 7+\sum _q2\right) \nonumber \\&\quad -\, 2 (\mathrm{Tr}[T_aT_cT_bT_d]+ \mathrm{Tr}[T_aT_dT_bT_c])\left( 24+\sum _q5\right) \nonumber \\&\quad +\,4(\mathrm{Tr}[T_aT_bT_cT_d]+ \mathrm{Tr}[T_aT_dT_cT_b])\left( 24+\sum _q5\right) , \nonumber \\&C_{abcd}^{(3)} = \delta _{ad}\delta _{bc}+\delta _{ac}\delta _{bd}+\delta _{ab}\delta _{cd} \nonumber \\&\quad -\, 6 (\mathrm{Tr}[T_aT_bT_cT_d] + \mathrm{Tr}[T_aT_dT_cT_b])\left( 7+\sum _q2\right) \nonumber \\&\quad -\, 2 (\mathrm{Tr}[T_aT_cT_bT_d]+ \mathrm{Tr}[T_aT_dT_bT_c])\left( 24+\sum _q5\right) \nonumber \\&\quad +\,4(\mathrm{Tr}[T_aT_bT_dT_c]+ \mathrm{Tr}[T_aT_cT_dT_b]) \left( 24+\sum _q5\right) .\quad \end{aligned}$$

In addition, the new $R_2$ counterterms involving external vector-like quarks are given by11In the conventions of Eqs. (9) and (11), $c_i$, $\mu _i$, and $p_i$ indicate the colour index (which can be associated either with the adjoint or the fundamental representation of *SU*(3)), the Lorentz index, and the four-momentum of the *i*th particle incoming to the $R_2^{\ldots i\ldots }$ vertex, respectively. Moreover, an explicit summation upon *q*, *Q* and *f* implies a summation over all quark species, the extra quark species and the Standard Model quark species, respectively.

In the phenomenological study undertaken in Sect. 3, the virtual one-loop contributions to the NLO predictions are evaluated with the MadLoop module [23], and then combined with the real contributions by means of the FKS subtraction method [24] as implemented in the MadFKS package [25]. Both MadLoop and MadFKS being part of MadGraph5_aMC@NLO [18], the entire calculation is entirely automated from the knowledge of the bare Lagrangian of Eq. (1) and the specification of the process of interest [30]. Technically, the translation of the model Lagrangian into a UFO library [22] that contains ultraviolet and $R_2$ counterterms and that could be used by MadGraph5_aMC@NLO is automatically performed with the FeynRules [19] and NLOCT [20] packages, the latter program taking care of the calculation of the one-loop ingredients of the model files. The corresponding FeynRules and UFO models have been made publicly available and can be downloaded from the webpage http://feynrules.irmp.ucl.ac.be/wiki/NLOModels.

### Benchmark scenarios

Throughout our phenomenological analysis, we adopt several series of benchmark scenarios in which one single vector-like quark is light enough so that it could be reachable at the LHC. Moreover, for the sake of simplicity, we enforce its decay to proceed via a single channel. We denote each class of scenarios by the acronym **QVi** where the symbol **Q** can be either *T*, *B*, *X* or *Y* and refers to the nature of the relevant extra quark, the symbol **V** refers to the nature of the gauge boson which the vector-like quark *Q* decays into and **i** is a generation number related to the family which the quark *Q* mixes with. For instance, the scenario **TW2** would correspond to a setup in which the Standard Model is supplemented by an extra up-type quark *T* that decays into a final state made of a *W*-boson and a strange quark with a branching ratio equal to 1.

These types of scenarios are motivated by several considerations. The mixings of the extra quark with the Standard Model sector are severely constrained by flavour-changing neutral current probes [17, 31, 32], LEP data [33, 34] and atomic parity violation measurements [35]. Taking the parameterisation of the $$\kappa $$, $$\tilde{\kappa }$$ and $$\hat{\kappa }$$ parameters of Eq. (3), sizeable mixings with all three generations are only allowed when the $$\kappa _{\scriptscriptstyle Q}$$ parameters are below $$10^{-2} $$–$$ 10^{-3}$$ [17]. Those bounds can, however, be relaxed when the mixing pattern is restricted to involve one or two quark generations. In our study, we enforce the vector-like quark mixing to only deal with one specific generation of Standard Model quarks, and we fix the values of the $$\kappa _{\scriptscriptstyle Q}$$ parameters to their current experimental limits of 0.07, 0.2 and 0.1 for mixings involving the first, second and third generation, respectively.

**Fig. 1:**
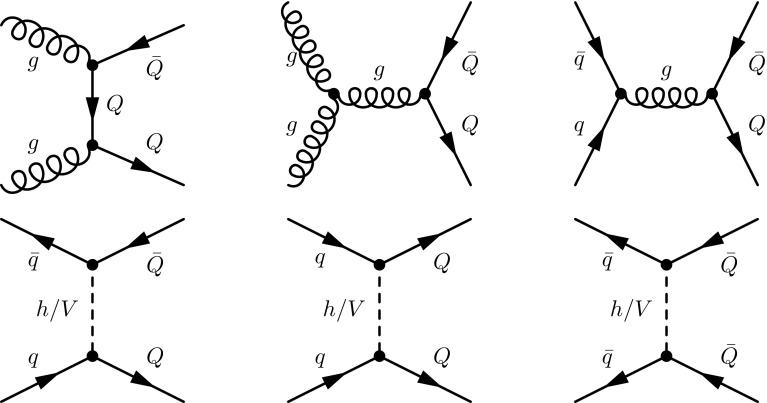
Strong (*upper*) and electroweak (*lower*) tree-level diagram contributions to the production of a pair of vector-like quarks, possibly carrying the same electric charge. The corresponding loop and real-emission diagrams are automatically generated by MadGraph5_aMC@NLO and contains one extra power of the strong coupling

## LHC phenomenology

In this section, we compute total cross sections and differential distributions both at the LO and NLO accuracy for several processes involving vector-like quarks. We study the genuine effects of the NLO corrections, as well as the induced reduction of the theoretical uncertainties. We then investigate the effects of matching fixed-order calculations to parton showers, both at LO and NLO. In Sects. 3.1 and 3.2, we, respectively, focus on vector-like quark pair and single production. For these processes, we consider QCD and electroweak diagrams that arise from the Lagrangian of Eq. (1), and we calculate the NLO effects for both contributions and their possible interferences. In Sect. 3.3, we focus on a set of additional processes where vector-like quark effects could be relevant. We consider the production of a single vector-like quark in association with a Standard Model weak or Higgs boson, and of a pair of Standard Model Higgs or weak bosons in the presence of vector-like quarks.

For the considered processes, the central (total and differential) cross-section values are computed after setting the renormalisation and factorisation scales to the average transverse mass of the final-state particles and by using the NLO set of the NNPDF 3.0 parton density functions (PDF) [36] accessed via the LHAPDF 6 library [37]. Scale uncertainties are derived by varying both scales independently by a factor of two up and down, and the PDF uncertainties are extracted following the recommendations of Ref. [38], and both contributions to the theoretical uncertainties are added in quadrature.

### Vector-like quark pair production at the LHC

Vector-like quark pair production is in general dominated by QCD contributions, which has the advantage to be independent of the model details. Model-dependent electroweak diagrams induced by the last four lines of the Lagrangian of Eq. (1) may, however, be non-negligible, in particular when the final state of interest can be produced from the scattering of one or two valence quarks. We focus on the production of a pair of vector-like quarks including the cases where they have the same electric charge,

12 $$\begin{aligned} p p \rightarrow Q \bar{Q},\quad p p \rightarrow Q Q\quad \text {and}\quad p p \rightarrow \bar{Q} \bar{Q}, \end{aligned}$$

with *Q* being either *T*, *B*, *X* or *Y*. While the first of these three subprocesses receives both strong (diagrams of the first line of Fig. 1) and electroweak (first diagram of the second line of Fig. 1) contributions, the latter two subprocesses can only be mediated by the *t*-channel exchange of a weak or Higgs boson (last two diagrams of the second line of Fig. 1).

NLO corrections to the strong contributions to the production of a pair of vector-like quarks (the diagrams of the first line of Fig. 1) can be automatically calculated within the MadGraph5_aMC@NLO framework. Thanks to the upgrading of the model to NLO as explained in the previous section, it is now sufficient to type in the program shell, taking the example of $T\bar{T}$ production, 
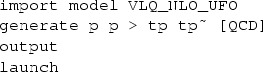
 With this set of instructions, we first import the UFO model associated with the Lagrangian of Eq. (1) and then start the calculation of the cross section and the generation of Monte Carlo events, at the NLO accuracy in QCD, for the production of a pair of $T\bar{T}$ quark and antiquark (whose UFO names are +tp+ and +tp +). Other vector-like quark processes can be obtained by replacing the +tp+ symbol by +bp+ (for a *B* quark), +x+ (for an *X* quark) and +y+ (for a *Y* quark). LO event generation can be achieved in the same way once the +[QCD]+ tag is omitted. We recall that the syntax is case insensitive.

For the electroweak channels (the diagrams of the second line of Fig. 1), the command to be typed in reads 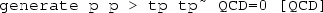 still for the same example of $T\bar{T}$ production. Other channels (including the production of a pair of heavy quarks carrying the same electric charge) can be addressed similarly. Care must, however, be taken as mixed electroweak and QCD loops appear at NLO. In our approach, we focus on NLO calculations in QCD, and not in QED or in the context of the electroweak theory. As a result, MadGraph5_aMC@NLO automatically discards Feynman diagrams tagged as an electroweak correction to a QCD graph, although in our case all diagrams should be kept for a proper cancellation of all divergences. One possible way to cure this issue would be to include in the UFO model library all ultraviolet and $R_2$ counterterms that would be necessary for undertaking mixed NLO calculations in QCD and QED and to implement in MadFKS the necessary subtraction terms. This, however, goes beyond the scope of this work, so that in order to maintain automation from the user standpoint, we have instead released a public script that should be called for diagram generation in order to prevent MadGraph5_aMC@NLO from discarding any loop diagram that would be tagged as an electroweak correction to a QCD Born diagram. More precisely, the script allows for the inclusion of all box diagrams containing at least two strong interaction vertices, but it removes weak-boson or Higgs-boson loop contributions that consist of an electroweak correction to a QCD Born process. Additionally, diagrams exhibiting the *t*-channel exchange of a weak or Higgs boson but with an additional gluon are kept. Not using the script instead yields the removal of several necessary box diagram contributions.

Finally, QCD and electroweak diagrams can interfere. Because of the mixing of QCD and electroweak interaction orders at the one-loop level and the missing counterterms and subtraction terms that have been mentioned above, MadGraph5_aMC@NLO cannot currently be used for the calculation of these interferences beyond the LO accuracy. We therefore rely on LO simulations and reweight instead the results to include a *K*-factor approximating the effect of the QCD corrections, denoted by $K_\mathrm{NLOQCD}^{(\mathrm int)}$, taken as the ratio of the NLO to the LO (differential) results. We choose this $K_\mathrm{NLOQCD}^{(\mathrm int)}$ factor to be the geometrical average of the $K_\mathrm{NLOQCD}^{(\mathrm QCD)}$ and $K_\mathrm{NLOQCD}^{(\mathrm EW)}$ *K*-factors obtained in the context of the QCD and electroweak diagrams taken independently,

13 $$\begin{aligned} K_\mathrm{NLOQCD}^{(\mathrm int)} = \sqrt{K_\mathrm{NLOQCD}^{(\mathrm QCD)}\ K_\mathrm{NLOQCD}^{(\mathrm EW)}}, \end{aligned}$$

where our notations explicitly indicate the nature of the corresponding underlying Born process. We have additionally checked that, for the central value, our procedure yields numerical differences that are of at most 2%. The related MadGraph5_aMC@NLO command allowing for generating interference events is, again for the example of $T\bar{T}$ production, 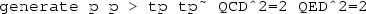 which is a standard command for LO event generation in MadGraph5_aMC@NLO.

All MadGraph5_aMC@NLO scripts necessary for differential and total cross-section calculations and event generation are available from the webpage http://feynrules.irmp.ucl.ac.be/wiki/NLOModels, together with the UFO and FeynRules models.

**Fig. 2:**
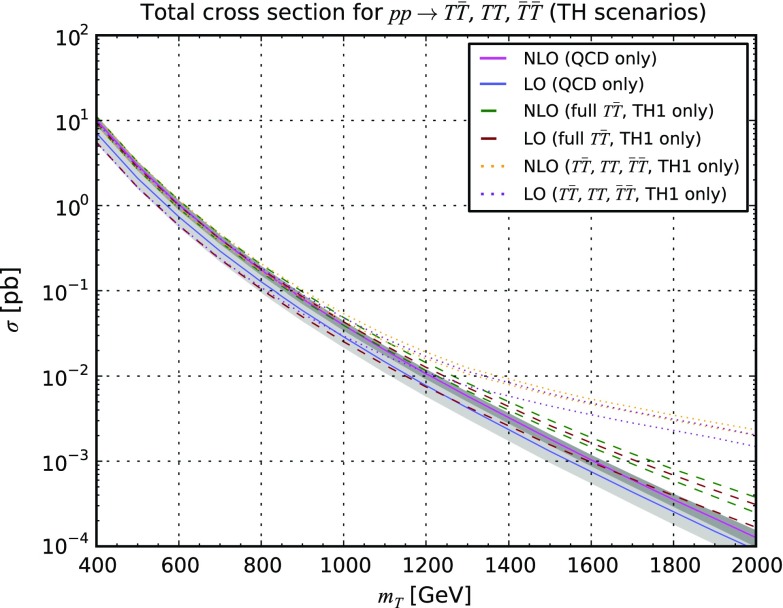
LO and NLO QCD inclusive cross sections for $T\bar{T}$/*TT*/$\bar{T} \bar{T}$ pair production at the LHC with $\sqrt{s}=13$ TeV. The QCD contribution results are presented together with the associated theoretical uncertainty bands, and we indicate the shifts in the bands that are induced by including weak or Higgs-boson exchange diagram contributions when they are non-negligible, which is only the case for the scenario **TH1**

In Fig. 2 and Table 1, we present LO and NLO total cross sections for the production of a pair of vector-like *T* quarks for the different scenarios introduced in Sect. 2.2, and we depict the dependence of the cross sections on the vector-like quark mass. We first focus on the pure QCD contribution that is independent of the vector-like quark nature (diagrams of the upper line of Fig. 1). The genuine NLO contributions are found to be important as they first induce a shift in the cross section of about 50% within the entire probed $m_{\scriptscriptstyle Q}$ range and next reduce the dependence of the rate on the unphysical factorisation and renormalisation scales. The uncertainty band is indeed significantly reduced when NLO effects are accounted for, the scale dependence being reduced to the level of about $\pm 10\%$ over the entire mass range. At the NLO, the scale dependence induced by the virtual contributions indeed partially compensates for the one stemming from the Born and real-emission diagrams. Our results for the pure QCD case (strong production Born diagrams only) agree with the literature, and we recall that this corresponds to the production of a top-quark pair with a different top-quark mass [39]. It is in contrast the first calculation including the impact of the electroweak diagrams at the NLO accuracy in QCD.

**Table 1:** LO and NLO QCD inclusive cross sections for $T\bar{T}$/*TT*/$\bar{T}\bar{T}$ production at the LHC, running at a center-of-mass energy of $\sqrt{s}=13$ TeV. The results are shown together with the associated scale and PDF relative uncertainties. For all scenarios but the **TH1** one, the predictions match the pure QCD results

$m_{\scriptscriptstyle T}$ [GeV]	Scenario	$\sigma _\mathrm{LO}$ [pb]	$\sigma _\mathrm{NLO}$ [pb]
400	QCD	$(7.069\ 10^{0}){}^{+32.0\%}_{-22.6\%}{}^{+2.7\%}_{-2.7\%}$	$(1.004\ 10^{1}){}^{+9.4\%}_{-11.3\%}{}^{+2.5\%}_{-2.5\%}$
	**TH1**	$(7.022\ 10^{0}){}^{+30.2\%}_{-23.8\%}{}^{+1.2\%}_{-4.1\%}$	$(9.980\ 10^{0}){}^{+8.0\%}_{-12.5\%}{}^{+1.2\%}_{-3.8\%}$
800	QCD	$(1.261\ 10^{-1}){}^{+33.2\%}_{-23.2\%}{}^{+3.8\%}_{-3.8\%}$	$(1.733\ 10^{-1}){}^{+8.5\%}_{-11.1\%}{}^{+4.4\%}_{-4.4\%}$
	**TH1**	$(1.244\ 10^{-1}){}^{+18.8\%}_{-31.2\%}{}^{+-7.3\%}_{-14.0\%}$	$(1.702\ 10^{-1}){}^{+-2.3\%}_{-20.0\%}{}^{+-6.0\%}_{-13.9\%}$
1200	QCD	$(7.685\ 10^{-3}){}^{+34.0\%}_{-23.7\%}{}^{+5.8\%}_{-5.8\%}$	$(1.061\ 10^{-2}){}^{+8.8\%}_{-11.4\%}{}^{+5.8\%}_{-5.8\%}$
	**TH1**	$(1.053\ 10^{-2}){}^{+-1.7\%}_{-36.7\%}{}^{+-18.4\%}_{-25.8\%}$	$(1.372\ 10^{-2}){}^{+-16.6\%}_{-29.0\%}{}^{+-18.2\%}_{-25.8\%}$
1600	QCD	$(7.477\ 10^{-4}){}^{+34.9\%}_{-24.2\%}{}^{+8.5\%}_{-8.5\%}$	$(1.030\ 10^{-3}){}^{+9.0\%}_{-11.6\%}{}^{+8.6\%}_{-8.6\%}$
	**TH1**	$(3.395\ 10^{-3}){}^{+-3.3\%}_{-27.0\%}{}^{+-13.3\%}_{-19.9\%}$	$(4.117\ 10^{-3}){}^{+-14.6\%}_{-21.8\%}{}^{+-14.4\%}_{-20.9\%}$
2000	QCD	$(8.980\ 10^{-5}){}^{+35.5\%}_{-24.5\%}{}^{+18.3\%}_{-18.3\%}$	$(1.260\ 10^{-4}){}^{+8.7\%}_{-11.7\%}{}^{+17.8\%}_{-17.8\%}$
	**TH1**	$(1.563\ 10^{-3}){}^{+4.2\%}_{-20.0\%}{}^{+-5.4\%}_{-13.0\%}$	$(1.960\ 10^{-3}){}^{+-6.3\%}_{-14.0\%}{}^{+-6.0\%}_{-13.6\%}$

In the considered scenarios, electroweak contributions to the production of a pair of vector-like quarks possibly carrying the same electric charge are found to be important only for the **TH1** scenario, the associated results being shown on Fig. 2 as dashed and dotted bands. These bands are, respectively, related to the production of a pair of vector-like quark–antiquark (dashed) and to the production of a pair of heavy quark regardless of their electric charge (dotted). In this last case, the contributions from the three processes of Eq. (12) are summed over. The **TH1** scenario features an extra quark that mixes with the first generation of Standard Model quarks so that parton density effects could lead to an enhancement of the production rate due to quark–antiquark, quark–quark and antiquark–antiquark (electroweak) scattering diagrams involving one or two initial valence quarks. This is particularly pronounced for setups featuring heavy vector-like quarks that require one to probe large Bjorken-*x* phase-space regions. As a consequence, the central cross-section values and the scale and parton density uncertainties are different from the pure QCD context since non-QCD diagrams (featuring a different initial state) dominate. This is illustrated in Table 1 for a few mass choices. Whereas the production rates are always larger, the uncertainties can be either smaller or larger than in the QCD case.

We observe a huge gain in cross section for **TH1** scenarios with a very heavy extra quark. This stems from Eq. (3) that shows that the coupling of the extra quark to the Higgs boson and a lighter quark has been taken proportional to the vector-like quark mass, and is thus enhanced for large values of $m_{\scriptscriptstyle Q}$. In principle, such a coupling should also be proportional to the related mixing matrix element $\zeta$ that compensates this enhancement, as shown in Eq. (3) and in Eq. (4). Setting $\zeta =1$, this feature is translated into the adopted value for the $\kappa _Q$ parameters.

Similar properties can be found in the context of the production of *B*, *X* and *Y* vector-like quarks, as illustrated in Appendix A.

**Fig. 3:**
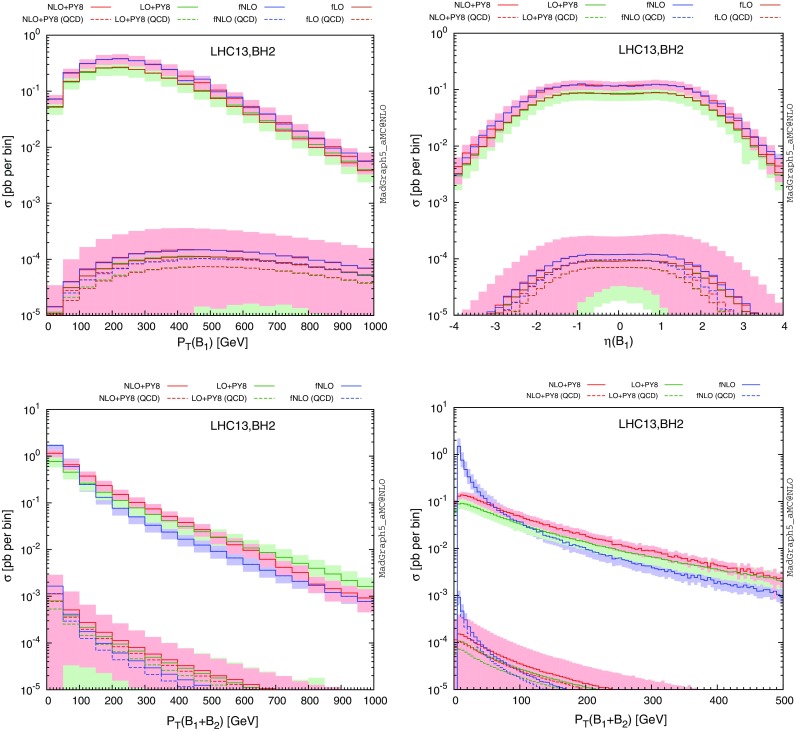
Properties of the pair-produced vector-like *B*-quarks. We present the transverse-momentum (*upper left*) and pseudorapidity (*upper right*) distribution for the first *B*-quark, as well as the transverse-momentum spectrum of the *B*-quark pair (*lower left* for the [0, 1000] GeV transverse-momentum region and *lower right* for a zoom in the [0, 500] GeV region). We compare fixed-order QCD predictions at the LO accuracy (*purple dashed curve*), NLO accuracy (*blue dashed curve*), and after matching these two calculations to parton showers (*green* and *red dashed curves* for the LO and NLO cases, respectively), and the *solid lines* depict the results once the electroweak diagram contributions are included. We have fixed the *B*-quark mass either to 500 GeV (*upper series of curves*) or to 1500 GeV (*lower series of curves*)

**Fig. 4:**
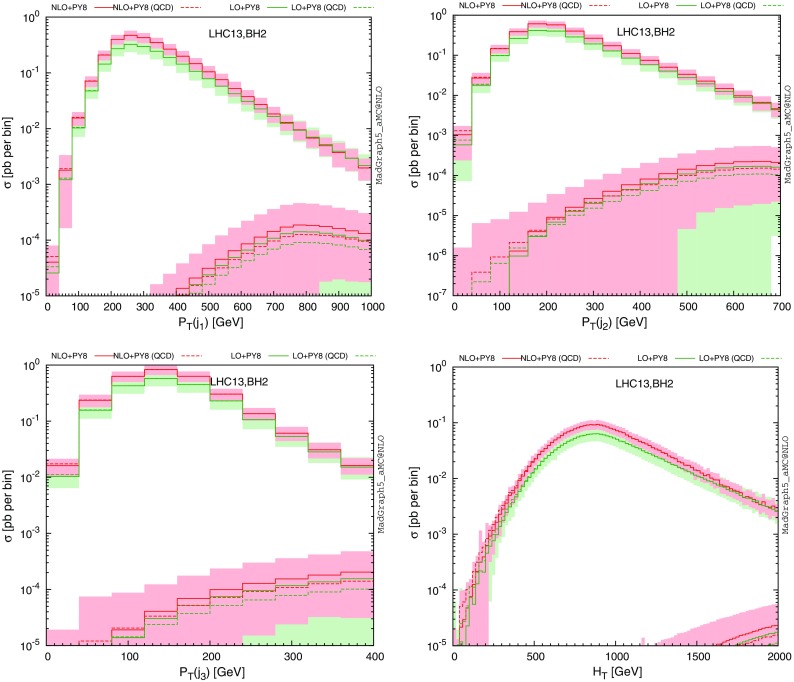
Same as in Fig. 3 but for the distribution in the transverse momentum of the first three leading jets and the $H_T$ variable defined as the sum of the transverse momentum of all the final-state jets and leptons

Accurate differential distributions are often helpful for setting more precise exclusion limits and refine the experimental search strategies. Our implementation in the MadGraph5_aMC@NLO platform can be used to this aim, and we present in Fig. 3 several differential distributions in several observables, including NLO and parton-shower effects. We have chosen the **BH2** class of benchmark scenarios with a vector-like quark mass set either to 500 GeV (upper series of curves on the figure) or 1500 GeV (lower series of curves on the figure). In our calculations, we have made use of the MadSpin [40] and MadWidth [41] programs for automatically taking care of the heavy quark decays in a way in which both off-shell and spin correlation effects are retained, matched the fixed-order calculation with the parton-shower and hadronisation infrastructure as modelled by the Pythia 8 package [42], and we have reconstructed all final-state jets by means of the anti-$k_T$ algorithm [43] with a radius parameter set to 0.5 as implemented in FastJet [44]. As shown on Figs. 3 and 4, our predictions confirm the total cross-section findings of Table 4 (see Appendix A). The contributions of the electroweak diagrams are, respectively, negligible and significant for light and heavy vector-like quarks. Moreover, the parton density uncertainties dominate for setups exhibiting a large $M_{\scriptscriptstyle B}$ value, rendering the theoretical predictions barely reliable.

In the two upper panels of Fig. 3, we study the properties of the first produced *B*-quark and show its transverse momentum $p_T(B_1)$ and pseudorapidity $\eta (B_1)$ distributions. In the case of a light *B*-quark, the parton-shower effects (green and red solid lines) slightly affect the shapes of the spectra predicted by the fixed-order calculations (blue and brown solid lines), both at the LO and NLO accuracies. For heavier vector-like quarks, slight modifications can be observed in the small $p_T$ and large $|\eta |$ region although the accurate modelling of the first extra jet does not yield any impact at the level of the individual *B*-quarks. These differences are largely covered by the theoretical uncertainties stemming from the poor knowledge of the parton densities in the relevant phase-space regions. These PDF uncertainties are dominant so that the reduction at the level of the scale uncertainties has a small impact. However, PDF uncertainties are expected to improve in the coming years thanks to new LHC data, so that it is mandatory to have NLO calculations available to get a better control on the predictions. In contrast, parton-shower effects are directly visible when distributions related to the two *B*-quarks considered as a pair are considered (Fig. 3). Focusing on the related transverse-momentum distributions, the fixed-order predictions (for which only the $p_T(B_1+B_2)=0$ bin is populated at LO) diverge at small $p_T$ due to soft and collinear radiation giving rise to large logarithms that must be resummed to all orders to obtain reliable predictions. This resummation is effectively achieved by matching the fixed-order calculations to parton showers, and the resulting distribution exhibits a reliable behaviour with a peak for $p_T(B_1+B_2)$ of about 10–20 GeV. Uncertainties originating from the choice of the shower algorithm and its inherent free parameters are, however, not estimated.

The magnitude of the electroweak diagrams is also studied (dashed lines). In the case of lighter *B*-quarks, the theoretical predictions are essentially driven by the QCD contributions so that no differences can be noticed. This contrasts with the heavy *B*-quark case where electroweak diagrams enhance the total rate by about 30% (see Appendix A) and also impact the differential distributions both in terms of normalisation and shape. This originates from the different topologies of the electroweak diagrams that feature a *t*-channel colourless boson exchange.

**Fig. 5:**
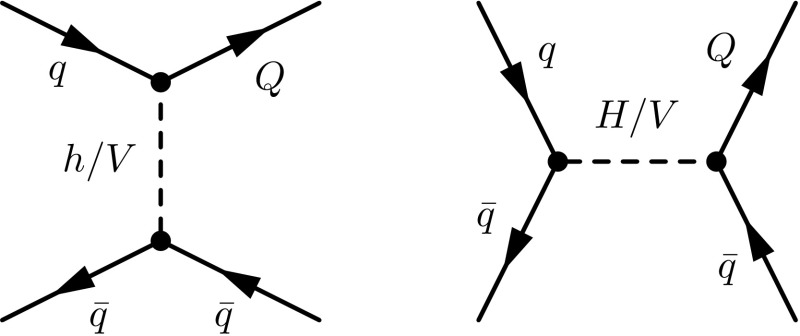
Representative Feynman diagram for single vector-like quark production in association with a quark. Other diagrams exist for final-state antiquarks, and all flavour assignments are understood. Virtual- and real-emission diagrams necessary for QCD correction calculations can be generated by MadGraph5_aMC@NLO

**Fig. 6:**
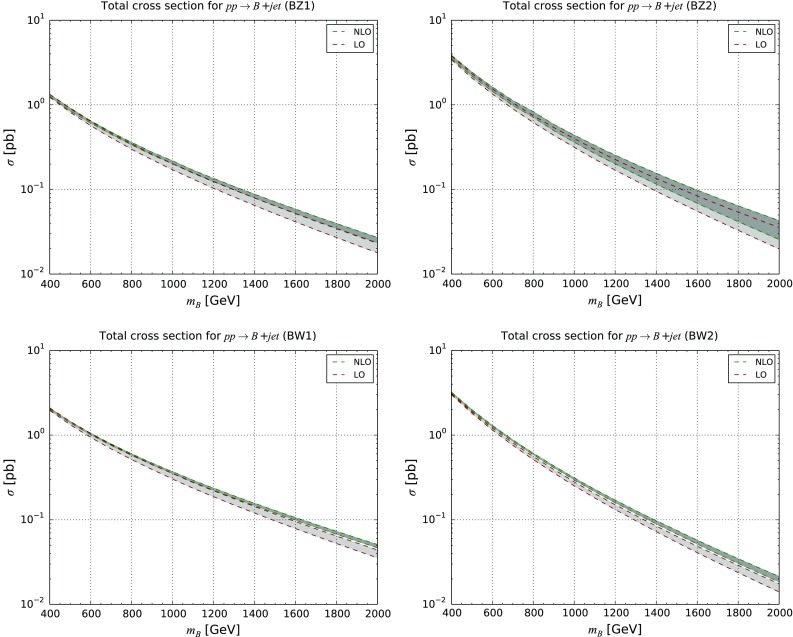
LO and NLO QCD inclusive cross sections for single *B* quark production at the LHC with $\sqrt{s}=13$ TeV. The results are presented together with the associated theoretical uncertainty bands for the **BZ1** (*upper left*), **BZ2** (*upper right*), **BW1** (*lower left*) and **BW2** (*lower right*) scenarios

We now include the vector-like quark decays into a Higgs boson and a strange quark and reconstruct the final-state jets as detailed above. Considering only hard central jets for which $|\eta |<2.4$ and $p_T>30$ GeV, we present the transverse-momentum distributions of the three leading jets as well as the spectrum of the $H_T$ variable defined as the scalar sum of the transverse momenta of all final-state jets and leptons in Fig. 4, the generated events being inclusive in the Higgs-boson decays. Leptons are included only if their transverse momentum is larger than 30 GeV, their pseudorapidity smaller than 2.4 in absolute value and if they are isolated from any jet by an angular distance in the transverse plane $\varDelta R$ of at least 0.5. Focusing on fixed-order predictions matched to parton showers, we observe that the first two leading jets are in general hard, as they result from the decay of two heavy coloured particles. In contrast, the structure of the $p_T$ dependence of the third jet is more representative of the one expected from a radiation jet, this jet being most of the time arising from initial-state or final-state radiation. Turning to the $H_T$ distribution (lower right panel of the figure), one notices a peak for very small $H_T$ values when the *B*-quark mass is fixed at 500 GeV. This feature arises from events where the two jets originating from the heavy quark decays are mis-reconstructed, the leading jet being thus the radiation jet so that the associated jet activity in the events is not significantly large.

**Table 2:** LO and NLO QCD inclusive cross sections for single *B* production at the LHC, running at a center-of-mass energy of $\sqrt{s}=13$ TeV. The results are shown together with the associated scale and PDF relative uncertainties in the context of several benchmark scenarios

$m_{\scriptscriptstyle B}$ [GeV]	Scenario	$\sigma _\mathrm{LO}$ [pb]	$\sigma _\mathrm{NLO}$ [pb]
400	**BZ1**	$(1.277\ 10^{0}){}^{+2.9\%}_{-3.0\%}{}^{+2.6\%}_{-2.6\%}$	$(1.288\ 10^{0}){}^{+0.9\%}_{-0.4\%}{}^{+2.6\%}_{-2.6\%}$
	**BZ2**	$(3.573\ 10^{0}){}^{+0.8\%}_{-1.3\%}{}^{+4.8\%}_{-4.8\%}$	$(3.668\ 10^{0}){}^{+1.0\%}_{-0.3\%}{}^{+4.7\%}_{-4.7\%}$
	**BW1**	$(2.021\ 10^{0}){}^{+2.9\%}_{-3.0\%}{}^{+1.7\%}_{-1.7\%}$	$(2.033\ 10^{0}){}^{+0.9\%}_{-0.6\%}{}^{+1.7\%}_{-1.7\%}$
	**BW2**	$(3.037\ 10^{0}){}^{+0.0\%}_{-0.7\%}{}^{+1.4\%}_{-1.4\%}$	$(3.161\ 10^{0}){}^{+1.3\%}_{-0.7\%}{}^{+1.4\%}_{-1.4\%}$
800	**BZ1**	$(3.197\ 10^{-1}){}^{+6.6\%}_{-5.9\%}{}^{+3.3\%}_{-3.3\%}$	$(3.461\ 10^{-1}){}^{+1.0\%}_{-1.2\%}{}^{+3.2\%}_{-3.2\%}$
	**BZ2**	$(6.777\ 10^{-1}){}^{+4.9\%}_{-4.6\%}{}^{+8.0\%}_{-8.0\%}$	$(7.671\ 10^{-1}){}^{+1.4\%}_{-1.3\%}{}^{+7.4\%}_{-7.4\%}$
	**BW1**	$(5.450\ 10^{-1}){}^{+6.4\%}_{-5.8\%}{}^{+1.8\%}_{-1.8\%}$	$(5.858\ 10^{-1}){}^{+1.0\%}_{-1.1\%}{}^{+1.8\%}_{-1.8\%}$
	**BW2**	$(5.335\ 10^{-1}){}^{+3.4\%}_{-3.5\%}{}^{+2.0\%}_{-2.0\%}$	$(5.938\ 10^{-1}){}^{+1.4\%}_{-0.8\%}{}^{+1.8\%}_{-1.8\%}$
1200	**BZ1**	$(1.129\ 10^{-1}){}^{+8.8\%}_{-7.6\%}{}^{+4.2\%}_{-4.2\%}$	$(1.291\ 10^{-1}){}^{+1.7\%}_{-2.3\%}{}^{+4.0\%}_{-4.0\%}$
	**BZ2**	$(1.966\ 10^{-1}){}^{+7.2\%}_{-6.5\%}{}^{+12.6\%}_{-12.6\%}$	$(2.268\ 10^{-1}){}^{+1.7\%}_{-2.0\%}{}^{+11.8\%}_{-11.8\%}$
	**BW1**	$(2.021\ 10^{-1}){}^{+8.5\%}_{-7.3\%}{}^{+2.0\%}_{-2.0\%}$	$(2.298\ 10^{-1}){}^{+1.5\%}_{-2.1\%}{}^{+2.1\%}_{-2.1\%}$
	**BW2**	$(1.406\ 10^{-1}){}^{+5.7\%}_{-5.4\%}{}^{+3.3\%}_{-3.3\%}$	$(1.645\ 10^{-1}){}^{+1.8\%}_{-1.8\%}{}^{+3.1\%}_{-3.1\%}$
1600	**BZ1**	$(4.607\ 10^{-2}){}^{+10.3\%}_{-8.8\%}{}^{+5.2\%}_{-5.2\%}$	$(5.519\ 10^{-2}){}^{+2.6\%}_{-3.1\%}{}^{+4.9\%}_{-4.9\%}$
	**BZ2**	$(6.934\ 10^{-2}){}^{+8.9\%}_{-7.8\%}{}^{+18.9\%}_{-18.9\%}$	$(8.316\ 10^{-2}){}^{+2.5\%}_{-2.8\%}{}^{+17.6\%}_{-17.6\%}$
	**BW1**	$(8.603\ 10^{-2}){}^{+9.9\%}_{-8.5\%}{}^{+2.4\%}_{-2.4\%}$	$(1.022\ 10^{-1}){}^{+2.4\%}_{-3.0\%}{}^{+2.4\%}_{-2.4\%}$
	**BW2**	$(4.447\ 10^{-2}){}^{+7.4\%}_{-6.7\%}{}^{+5.1\%}_{-5.1\%}$	$(5.423\ 10^{-2}){}^{+2.5\%}_{-2.6\%}{}^{+4.7\%}_{-4.7\%}$
2000	**BZ1**	$(2.022\ 10^{-2}){}^{+11.6\%}_{-9.7\%}{}^{+6.4\%}_{-6.4\%}$	$(2.516\ 10^{-2}){}^{+3.3\%}_{-3.9\%}{}^{+6.1\%}_{-6.1\%}$
	**BZ2**	$(2.751\ 10^{-2}){}^{+10.2\%}_{-8.7\%}{}^{+26.9\%}_{-26.9\%}$	$(3.412\ 10^{-2}){}^{+3.2\%}_{-3.5\%}{}^{+25.1\%}_{-25.1\%}$
	**BW1**	$(3.922\ 10^{-2}){}^{+11.2\%}_{-9.4\%}{}^{+2.9\%}_{-2.9\%}$	$(4.873\ 10^{-2}){}^{+3.3\%}_{-3.8\%}{}^{+2.8\%}_{-2.8\%}$
	**BW2**	$(1.564\ 10^{-2}){}^{+8.8\%}_{-7.8\%}{}^{+7.3\%}_{-7.3\%}$	$(1.981\ 10^{-2}){}^{+3.2\%}_{-3.3\%}{}^{+6.9\%}_{-6.9\%}$

### Single vector-like quark production in association with jets

Single vector-like quark production mechanisms are of a pure electroweak nature. The associated predictions are thus model-dependent as the sizes of the electroweak vector-like quark couplings are free parameters of the model. Comparing vector-like quark single and pair production, the latter gets an enhancement originating from the presence of strong diagram contributions (first line of Fig. 1) together with a phase-space suppression for large vector-like quark mass values. In contrast, electroweak single vector-like quark production is less suppressed for large vector-like quark masses, which could compensate the weakness of the involved interaction vertices and make this channel the main LHC discovery mode for a heavy vector-like quark. As a results, several ATLAS and CMS vector-like quark searches also target their single-production mode [10, 45–48].

In this section, we focus on vector-like quark single production in association with jets,

14 $$\begin{aligned} p p \rightarrow Q j\ \text {or}\ \bar{Q} j \quad \text {with}\ Q = T, B, X \ \text {or}\ Y. \end{aligned}$$

Other single-production mechanisms exist, with, for instance, a final-state gauge or a Higgs boson, but we refer to Sect. 3.3 for the latter. A representative set of Feynman diagrams related to single vector-like quark production with jets is shown on Fig. 5, and NLO cross-section calculation and event generation can be achieved with MadGraph5_aMC@NLO by typing in the program interpreter the command 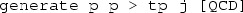 for single *T* production. Other processes with a different final-state vector-like quark can be accounted for with a similar syntax, and LO event generation only necessitates to remove the +[QCD]+ tag. As for electroweak contributions to vector-like quark pair production, mixed QCD and electroweak loop diagrams appear at the NLO level and must be treated consistently for getting ultraviolet-finite results. This step being not automated, we provide scripts that steer the event generation process on the UFO model webpage.

In Fig. 6 and Table 2, we present LO and NLO total cross section for single *B* quark production for the **BZ1**, **BZ2**, **BW1** and **BW2** scenarios introduced in Sect. 2.2 for which single vector-like *B* quark production occurs via *Z*-boson or *W*-boson exchanges. This consists of the first NLO predictions for a single vector-like quark production process. Although all depicted total cross sections exhibit a similar order of magnitude, a small hierarchy is observed. It is driven by an interplay of the parton densities that enhance mechanisms involving first generation quarks and of the new physics coupling strengths that are much larger for vector-like quark mixings with second generation ($\kappa _{\scriptscriptstyle Q}=0.2$) than with first generation quarks ($\kappa _{\scriptscriptstyle Q}=0.07$). For vector-like *B*-quark masses smaller than 500 GeV, single-production cross sections are slightly suppressed by a factor of 2 or 3 with respect to the strong production of a pair of *B* quarks (see Appendix A), but feature a similar order of magnitude for $m_{\scriptscriptstyle B} \in [500, 800]$ GeV. In contrast, single-*B* production always dominates for heavier vector-like quarks by up to two orders of magnitude for larger values of $m_{\scriptscriptstyle B}$. For scenarios with smaller $\kappa _{\scriptscriptstyle Q}$ values, the changes in the relative importance of the two production channels can, however, be shifted towards higher masses.

**Fig. 7:**
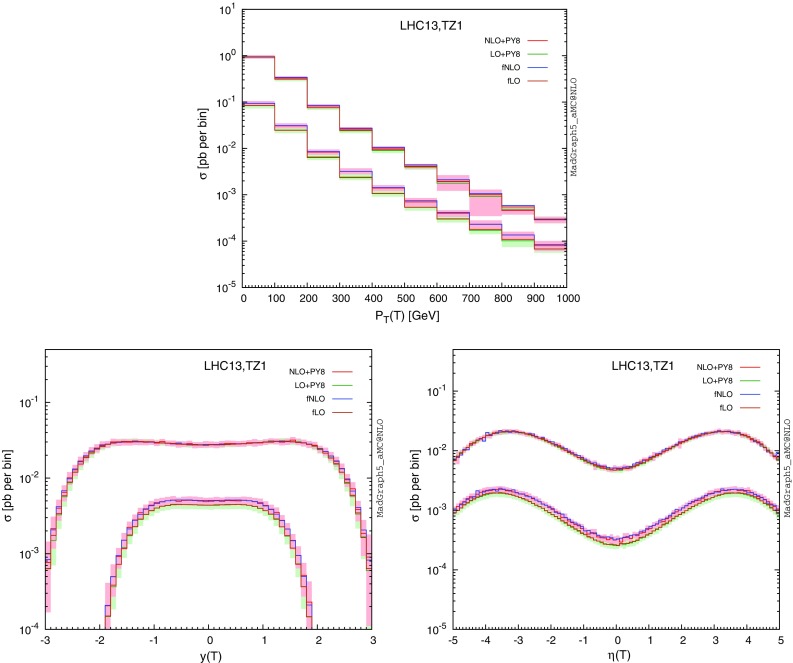
Differential distributions depicting the properties of a singly produced vector-like *T* (or $\bar{T}$) quark. We present its transverse momentum (*upper*), rapidity (*lower left*) and pseudorapidity (*lower right*) spectrum and compare fixed-order predictions at the LO accuracy (*purple curve*) and NLO accuracy (*blue curve*), as well as prediction including the matching of these two calculations to parton showers (*green* and *red curves* for the LO and NLO cases, respectively). We have fixed the heavy quark mass either to 500 GeV (*upper series of curves*) or to 1500 GeV (*lower series of curves*)

In Fig. 7, we turn to the study of differential distributions related to inclusive single *T*-quark production at the LHC, focusing on the **TZ1** scenario as an illustrative benchmark point and for a vector-like quark mass $m_{\scriptscriptstyle T}$ fixed to 500 GeV or 1500 GeV. Event generation and reconstruction is performed following the guidelines mentioned in Sect. 3.1, and we present the transverse momentum $p_T(T)$, rapidity *y*(*T*) and pseudorapidity $\eta (T)$ spectrum of the vector-like quark. We show predictions both at the fixed-order (purple and blue curves for the LO and NLO accuracy, respectively) and after matching the results to parton showers (green and red curves for the LO and NLO accuracy, respectively). Theoretical uncertainties originating from scale variations and parton densities are included for the matched predictions. In general, NLO effects only moderately affect the shape of the different spectra but in contrast drastically reduce the theoretical scale uncertainties and allow for a better prediction of the spectrum normalisation. Similarly to the pair-production case, matching to parton showers only mildly impacts fixed-order predictions for the properties of the produced heavy quarks. Regardless of $m_{\scriptscriptstyle T}$, the transverse-momentum distribution of singly produced *T* quarks exhibits a typical steeply falling behaviour with increasing values of $p_T(T)$ (with a peak at low $p_T$, which is invisible due to the binning choice), and the vector-like quark is quite forward by virtue of the process topology, with $|\eta |>2$ in average.

**Fig. 8:**
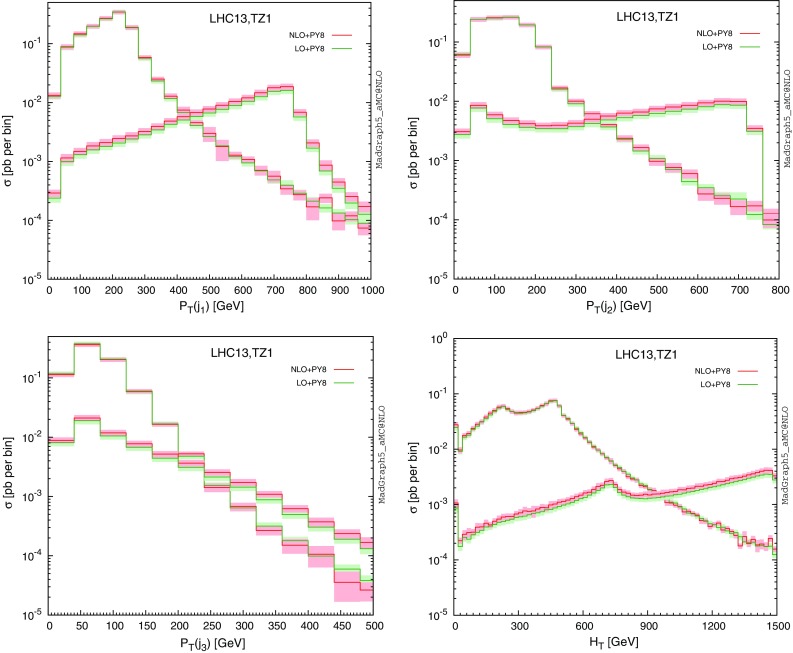
Differential distributions depicting the properties of the decay products of a singly produced vector-like *T* (or $\bar{T}$) quark. We present the transverse-momentum distributions of the three leading jets, as well as the one in the $H_T$ variable defined as the scalar sum of all jet and isolated lepton transverse momenta. We compare LO (*green*) and NLO (*red*) predictions after matching the fixed-order calculations to parton showers. We have fixed the heavy quark mass either to 500 GeV or to 1500 GeV

In Fig. 8, we study the properties of the decay products of the heavy quarks that consist of a *Z*-boson and an up quark in the **TZ1** scenario. We first present the transverse-momentum spectrum of the three leading jets with a pseudorapidity satisfying $|\eta |<2.5$ and a transverse-momentum larger than 30 GeV. Being inclusive in the *Z*-boson decay, the jets can originate either from the heavy *T*-quark decay, from the *Z*-boson decay or from initial- or final-state radiation. Focusing on the leading jet $p_T$ distribution, we observe that it peaks at half the *T*-quark mass, which shows that the leading jet is often issued from the heavy quark decay. In contrast, the second jet transverse-momentum distribution exhibit a plateau extending up to half the *T*-mass, which we conclude that it could alternatively originate either directly from the *T*-quark decay, or from the *Z*-boson decay. The third jet finally shows a different behaviour, the distribution peaking this time at a lower $p_T$ value, so that it is likely to be connected to the hard process.

In addition to the leading jet transverse-momentum distributions, we also present the distribution in the $H_T$ variable defined as the scalar sum of the transverse momentum of the final-state jets and isolated leptons, the latter being only considered if their pseudorapidity fulfils $\eta |<2.4$ and their transverse momentum $p_T>30$ GeV. Moreover, we require the leptons to be well separated from any jet, imposing the angular distance $\varDelta R$ to be larger than 0.5. The $H_T$ variable exhibits a non-trivial structure which peaks both at the *T*-quark mass $m_{\scriptscriptstyle T}$ and at $m_{\scriptscriptstyle T}/2$, the second peak arising from cases where the *Z*-boson decays invisibly.

In all cases, the shapes turn out to be only slightly affected by the NLO effects and the uncertainties are drastically reduced.

**Fig. 9:**
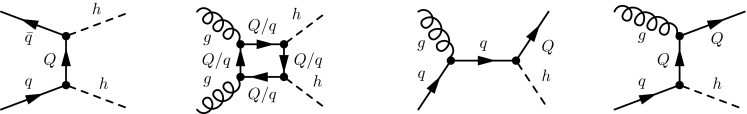
Representative Feynman diagram for Higgs-boson pair production that depend on vector-like quark exchange (two leftmost diagrams) and for single vector-like quark production in association with a Higgs boson (two rightmost diagrams). Similar diagrams exist when Higgs bosons are replaced by weak gauge bosons. With the second diagram, we include LO loop-induced contributions to Higgs-boson pair production that can only be calculated at the LO accuracy with MadGraph5_aMC@NLO

**Table 3:** LO and NLO QCD inclusive cross sections for Higgs-boson (upper) and *Z*-boson (lower) pair production at the LHC, running at a center-of-mass energy of $\sqrt{s}=13$ TeV. The results are shown together with the associated scale and PDF relative uncertainties in the context of the **TH1** (upper) and **TZ1** (lower) class of benchmark scenarios

$m_{\scriptscriptstyle T}$ [GeV]	Scenario	$\sigma _\mathrm{LO}$ [pb]	$\sigma _\mathrm{NLO}$ [pb]
400	**TH1**	$(1.254\ 10^{-4}){}^{+3.3\%}_{-3.3\%}{}^{+2.1\%}_{-2.1\%}$	$(1.883\ 10^{-4}){}^{+4.8\%}_{-4.1\%}{}^{+1.8\%}_{-1.8\%}$
800	**TH1**	$(1.043\ 10^{-4}){}^{+5.9\%}_{-5.3\%}{}^{+2.3\%}_{-2.3\%}$	$(1.815\ 10^{-4}){}^{+7.0\%}_{-5.9\%}{}^{+1.8\%}_{-1.8\%}$
1200	**TH1**	$(8.130\ 10^{-5}){}^{+7.3\%}_{-6.5\%}{}^{+2.7\%}_{-2.7\%}$	$(1.714\ 10^{-4}){}^{+8.9\%}_{-7.5\%}{}^{+1.7\%}_{-1.7\%}$
1600	**TH1**	$(6.092\ 10^{-5}){}^{+8.4\%}_{-7.3\%}{}^{+3.1\%}_{-3.1\%}$	$(1.600\ 10^{-4}){}^{+10.8\%}_{-9.0\%}{}^{+1.6\%}_{-1.6\%}$
2000	**TH1**	$(4.519\ 10^{-5}){}^{+9.1\%}_{-7.9\%}{}^{+3.6\%}_{-3.6\%}$	$(1.513\ 10^{-4}){}^{+12.4\%}_{-10.2\%}{}^{+1.6\%}_{-1.6\%}$
400	**TZ1**	$(1.017\ 10^{1}){}^{+4.7\%}_{-5.9\%}{}^{+1.5\%}_{-1.5\%}$	$(1.314\ 10^{1}){}^{+3.3\%}_{-3.9\%}{}^{+1.5\%}_{-1.5\%}$
800	**TZ1**	$(1.019\ 10^{1}){}^{+4.7\%}_{-5.9\%}{}^{+1.5\%}_{-1.5\%}$	$(1.312\ 10^{1}){}^{+3.3\%}_{-3.8\%}{}^{+1.5\%}_{-1.5\%}$
1200	**TZ1**	$(1.020\ 10^{1}){}^{+4.7\%}_{-5.8\%}{}^{+1.5\%}_{-1.5\%}$	$(1.312\ 10^{1}){}^{+3.3\%}_{-3.9\%}{}^{+1.5\%}_{-1.5\%}$
1600	**TZ1**	$(1.020\ 10^{1}){}^{+4.7\%}_{-5.9\%}{}^{+1.5\%}_{-1.5\%}$	$(1.313\ 10^{1}){}^{+3.2\%}_{-3.8\%}{}^{+1.5\%}_{-1.5\%}$
2000	**TZ1**	$(1.020\ 10^{1}){}^{+4.7\%}_{-5.9\%}{}^{+1.5\%}_{-1.5\%}$	$(1.313\ 10^{1}){}^{+3.3\%}_{-3.8\%}{}^{+1.5\%}_{-1.5\%}$

### Other processes impacted by the presence of vector-like quarks

Vector-like quarks can also affect Standard Model processes due to additional Feynman diagram contributions featuring a virtual heavy quark. For instance, the production of a pair of Higgs bosons could proceed via the new physics diagrams shown in Fig. 9, similar diagrams existing for the diboson case. MadGraph5_aMC@NLO can be used for event generation at the NLO accuracy, once topologies featuring intermediate heavy quark resonances are treated accordingly. This is achieved with the command
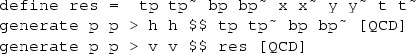


for di-Higgs and diboson production, respectively, using the $$ symbol to remove any possible intermediate resonance. In the last case, the pair of symbols v v stands either for w+ w- or z z according to the weak boson under consideration. LO event generation can finally be achieved by removing the +[QCD]+ tag.

Additional single vector-like quark production processes where the heavy quark is produced in association with a Standard Model boson can be considered as extra mechanisms useful for seeking for vector-like quarks (diagrams shown in the rightmost part of Fig. 9). Such processes can be simulated with MadGraph5_aMC@NLO, by typing in the commands 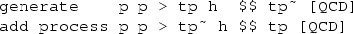 for $$T/\bar{T}$$ production, as an example. Once again, the $$ symbol is used in order to avoid intermediate resonances. *VQ* production can be undertaken similarly.

**Fig. 10:**
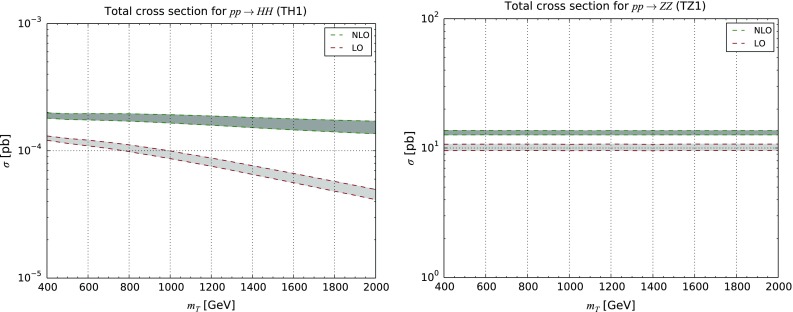
LO and NLO QCD inclusive cross sections for Higgs-boson (*left*) and *Z*-boson (*right*) pair production at the LHC with $\sqrt{s}=13$ TeV. The results are presented together with the associated theoretical uncertainty bands for the **TH1** (*left*) and **TZ1** (*right*) scenarios

As an illustrative example, we show in Table 3 and Fig. 10 total rates at LO and NLO for Higgs-boson (upper) and Z-boson (lower) pair production, respectively, for scenarios featuring a *T* quark interacting with the first generation of Standard Model quarks and either the Higgs boson or the *Z*-boson. We observe gigantic *K*-factors in the case of di-Higgs production. This enhancement is connected to an interplay of two effects. Turning back to the observations made in Sect. 3.1, the vector-like quark coupling to light quarks and a Higgs boson has strength that is proportional to the heavy quark mass, and thus becomes stronger and stronger with increasing vector-like quark masses. In addition, a new channel where the initial state is comprised of a gluon and a quark opens up at NLO. This component of the NLO cross section turns out to dominate due to the large gluon density in the proton. As a result, the total cross section for vector-like-quark-mediated di-Higgs production is more or less constant with the heavy quark mass at the NLO QCD accuracy, which contrasts with the LO case.

## Conclusions

We have modified a previously introduced model-independent parameterisation suitable for the study of the vector-like quark phenomenology at the LHC so that it is now suitable for NLO calculations in QCD matched to parton showers within the MadGraph5_aMC@NLO framework. We have illustrated its usage in the context of vector-like quark pair production and vector-like quark single production in association either with a jet or with a weak or Higgs boson. For all showcased processes, we have considered QCD and electroweak diagram contributions and investigated NLO and parton-shower effects on the normalisation and shapes of the associated kinematical distributions.

**Fig. 11:**
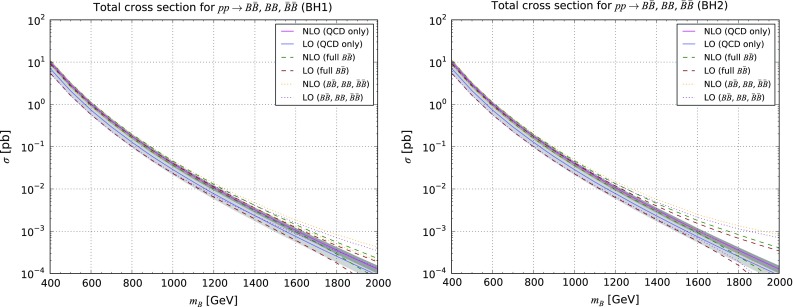
LO and NLO QCD inclusive cross sections for *B* pair production at the LHC, for $\sqrt{s}=13$ TeV. The QCD contribution results are presented together with the associated theoretical uncertainty bands, and we indicate the shifts in the bands that are induced by weak diagram contributions in a scenario in which they are non-negligible

**Table 4:** LO and NLO QCD inclusive cross sections for *B* pair production at the LHC, running at a center-of-mass energy of $\sqrt{s}=13$ TeV. The results are shown together with the associated scale and PDF relative uncertainties in the context of several benchmark scenarios. For all non-indicated scenarios, the results match the pure QCD one given in Table 1

$m_{\scriptscriptstyle B}$ [GeV]	Scenario	$\sigma _\mathrm{LO}$ [pb]	$\sigma _\mathrm{NLO}$ [pb]
400	**BH1**	$(7.025\ 10^{0}){}^{+31.0\%}_{-23.3\%}{}^{+1.5\%}_{-3.2\%}$	$(9.995\ 10^{0}){}^{+7.6\%}_{-11.5\%}{}^{+1.6\%}_{-3.1\%}$
	**BH2**	$(7.047\ 10^{0}){}^{+31.2\%}_{-23.1\%}{}^{+1.7\%}_{-3.0\%}$	$(1.011\ 10^{1}){}^{+9.8\%}_{-12.3\%}{}^{+2.0\%}_{-3.1\%}$
800	**BH1**	$(1.241\ 10^{-1}){}^{+26.2\%}_{-27.5\%}{}^{+-1.8\%}_{-9.1\%}$	$(1.715\ 10^{-1}){}^{+2.7\%}_{-15.5\%}{}^{+-1.1\%}_{-9.0\%}$
	**BH2**	$(1.282\ 10^{-1}){}^{+29.2\%}_{-25.0\%}{}^{+1.8\%}_{-7.2\%}$	$(1.749\ 10^{-1}){}^{+5.6\%}_{-13.1\%}{}^{+2.0\%}_{-7.1\%}$
1200	**BH1**	$(7.903\ 10^{-3}){}^{+16.2\%}_{-32.4\%}{}^{+-7.9\%}_{-16.9\%}$	$(1.088\ 10^{-2}){}^{+-4.8\%}_{-21.5\%}{}^{+-7.0\%}_{-16.9\%}$
	**BH2**	$(8.883\ 10^{-3}){}^{+23.0\%}_{-26.1\%}{}^{+6.7\%}_{-18.3\%}$	$(1.189\ 10^{-2}){}^{+2.1\%}_{-15.4\%}{}^{+5.6\%}_{-16.7\%}$
1600	**BH1**	$(1.016\ 10^{-3}){}^{+10.5\%}_{-31.3\%}{}^{+-6.8\%}_{-21.1\%}$	$(1.356\ 10^{-3}){}^{+-7.5\%}_{-22.3\%}{}^{+-7.6\%}_{-20.5\%}$
	**BH2**	$(1.255\ 10^{-3}){}^{+14.8\%}_{-24.3\%}{}^{+25.7\%}_{-41.5\%}$	$(1.643\ 10^{-3}){}^{+-1.7\%}_{-15.7\%}{}^{+22.3\%}_{-38.1\%}$
2000	**BH1**	$(2.138\ 10^{-4}){}^{+13.1\%}_{-24.3\%}{}^{+7.3\%}_{-24.0\%}$	$(2.817\ 10^{-4}){}^{+-3.1\%}_{-16.0\%}{}^{+5.3\%}_{-22.7\%}$
	**BH2**	$(3.262\ 10^{-4}){}^{+11.0\%}_{-19.4\%}{}^{+78.6\%}_{-91.1\%}$	$(4.133\ 10^{-4}){}^{+-2.1\%}_{-12.2\%}{}^{+69.7\%}_{-82.8\%}$

We have found that NLO *K*-factors are important, both globally (at the total-rate level) and at the differential distribution level and could hence potentially impact limits currently extracted from vector-like quark search results of the ATLAS and CMS collaborations. We have in particular noticed the existence of potentially huge *K*-factors for new physics scenarios involving the coupling of a heavy vector-like quark to first generation quarks and a Higgs boson due to new production channels that open at the NLO accuracy. This motivates further investigations, in particular to assess how an experimental analysis including detector effects could benefit from the gain in cross section stemming from the new topologies that dominate at NLO and to determine the impact on the current vector-like quark limits and LHC discovery potential.

## Figures and Tables

**Fig. 12:**
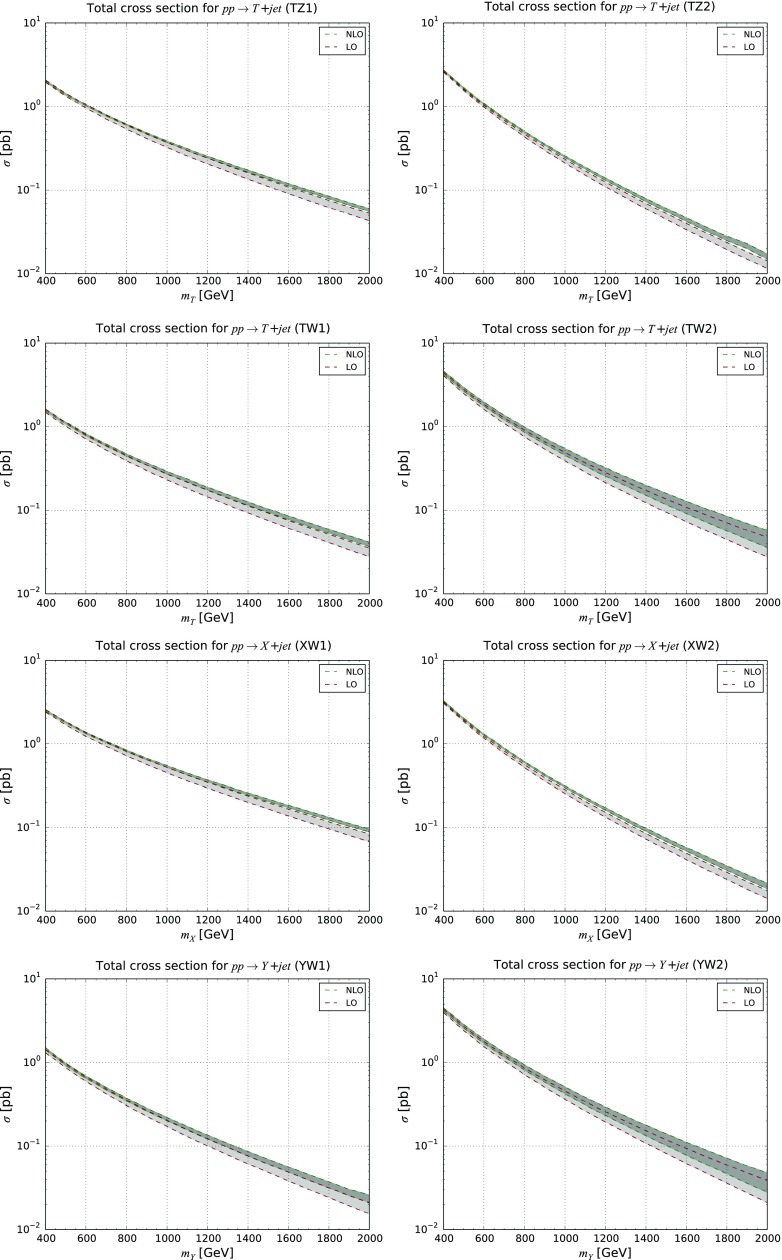
LO and NLO QCD inclusive cross sections for single *T*, *X* and *Y* quark production at the LHC with $\sqrt{s}=13$ TeV, for various benchmark scenarios

## Appendix A: Total cross sections for the production of a pair of *B*, *X* or *Y* quarks

In Fig. 11 and Table 4, we present LO and NLO total cross sections related to the production of a pair of vector-like *B* quarks for the different scenarios introduced in Sect. 2.2. We depict the dependence of the cross sections on the vector-like quark mass and recall that the pure QCD contributions can be found in Table 2 as they are independent of the vector-like quark nature. Results for *X* and *Y* pair production are not shown as non-QCD contributions are negligible for all considered scenarios.

## Appendix B: Total cross sections for the single production of a *T*, *X* or *Y* quark

In Fig. 12, Table 5 and Table 6, we present LO and NLO total cross sections related to the production of a single vector-like *T*, *X* and *Y* quark for different scenarios introduced in Sect. 2.2. In each case, we depict the dependence of the cross sections on the vector-like quark mass and study the uncertainties stemming from scale variation.

**Table 5:** LO and NLO QCD inclusive cross sections for single *T* production at the LHC, running at a center-of-mass energy of $\sqrt{s}=13$ TeV. The results are shown together with the associated scale and PDF relative uncertainties in the context of several benchmark scenarios

$m_{\scriptscriptstyle T}$ [GeV]	Scenario	$\sigma _\mathrm{LO}$ [pb]	$\sigma _\mathrm{NLO}$ [pb]
400	**TZ1**	$(1.995\ 10^{0}){}^{+2.6\%}_{-2.7\%}{}^{+1.6\%}_{-1.6\%}$	$(1.987\ 10^{0}){}^{+0.8\%}_{-0.6\%}{}^{+1.7\%}_{-1.7\%}$
	**TZ2**	$(2.613\ 10^{0}){}^{+0.1\%}_{-1.0\%}{}^{+1.2\%}_{-1.2\%}$	$(2.685\ 10^{0}){}^{+1.1\%}_{-0.6\%}{}^{+1.2\%}_{-1.2\%}$
	**TW1**	$(1.541\ 10^{0}){}^{+3.4\%}_{-3.3\%}{}^{+2.4\%}_{-2.4\%}$	$(1.575\ 10^{0}){}^{+0.9\%}_{-0.2\%}{}^{+2.4\%}_{-2.4\%}$
	**TW2**	$(4.229\ 10^{0}){}^{+1.1\%}_{-1.5\%}{}^{+4.5\%}_{-4.5\%}$	$(4.392\ 10^{0}){}^{+1.1\%}_{-0.3\%}{}^{+4.4\%}_{-4.4\%}$
800	**TZ1**	$(5.657\ 10^{-1}){}^{+6.2\%}_{-5.5\%}{}^{+1.8\%}_{-1.8\%}$	$(6.014\ 10^{-1}){}^{+0.9\%}_{-0.9\%}{}^{+1.8\%}_{-1.8\%}$
	**TZ2**	$(4.488\ 10^{-1}){}^{+3.2\%}_{-3.4\%}{}^{+1.8\%}_{-1.8\%}$	$(4.925\ 10^{-1}){}^{+1.3\%}_{-0.6\%}{}^{+1.7\%}_{-1.7\%}$
	**TW1**	$(4.183\ 10^{-1}){}^{+6.9\%}_{-6.1\%}{}^{+3.0\%}_{-3.0\%}$	$(4.560\ 10^{-1}){}^{+1.0\%}_{-1.4\%}{}^{+2.9\%}_{-2.9\%}$
	**TW2**	$(8.272\ 10^{-1}){}^{+5.0\%}_{-4.7\%}{}^{+7.7\%}_{-7.7\%}$	$(9.175\ 10^{-1}){}^{+1.1\%}_{-1.2\%}{}^{+7.2\%}_{-7.2\%}$
1200	**TZ1**	$(2.214\ 10^{-1}){}^{+8.2\%}_{-7.1\%}{}^{+1.9\%}_{-1.9\%}$	$(2.483\ 10^{-1}){}^{+1.4\%}_{-1.9\%}{}^{+2.0\%}_{-2.0\%}$
	**TZ2**	$(1.168\ 10^{-1}){}^{+5.6\%}_{-5.3\%}{}^{+3.2\%}_{-3.2\%}$	$(1.348\ 10^{-1}){}^{+1.6\%}_{-1.6\%}{}^{+2.9\%}_{-2.9\%}$
	**TW1**	$(1.572\ 10^{-1}){}^{+8.9\%}_{-7.6\%}{}^{+3.5\%}_{-3.5\%}$	$(1.812\ 10^{-1}){}^{+1.9\%}_{-2.4\%}{}^{+3.5\%}_{-3.5\%}$
	**TW2**	$(2.476\ 10^{-1}){}^{+7.3\%}_{-6.5\%}{}^{+12.0\%}_{-12.0\%}$	$(2.878\ 10^{-1}){}^{+2.0\%}_{-2.2\%}{}^{+11.3\%}_{-11.3\%}$
1600	**TZ1**	$(9.897\ 10^{-2}){}^{+9.6\%}_{-8.3\%}{}^{+2.2\%}_{-2.2\%}$	$(1.163\ 10^{-1}){}^{+2.1\%}_{-2.7\%}{}^{+2.1\%}_{-2.1\%}$
	**TZ2**	$(3.657\ 10^{-2}){}^{+7.3\%}_{-6.7\%}{}^{+4.9\%}_{-4.9\%}$	$(4.417\ 10^{-2}){}^{+2.3\%}_{-2.4\%}{}^{+4.5\%}_{-4.5\%}$
	**TW1**	$(6.774\ 10^{-2}){}^{+10.3\%}_{-8.8\%}{}^{+4.2\%}_{-4.2\%}$	$(8.151\ 10^{-2}){}^{+2.8\%}_{-3.3\%}{}^{+4.0\%}_{-4.0\%}$
	**TW2**	$(9.040\ 10^{-2}){}^{+9.0\%}_{-7.8\%}{}^{+17.7\%}_{-17.7\%}$	$(1.095\ 10^{-1}){}^{+2.6\%}_{-3.0\%}{}^{+16.6\%}_{-16.6\%}$
2000	**TZ1**	$(4.721\ 10^{-2}){}^{+10.9\%}_{-9.2\%}{}^{+2.4\%}_{-2.4\%}$	$(5.771\ 10^{-2}){}^{+2.9\%}_{-3.5\%}{}^{+2.4\%}_{-2.4\%}$
	**TZ2**	$(1.277\ 10^{-2}){}^{+8.7\%}_{-7.8\%}{}^{+7.0\%}_{-7.0\%}$	$(1.600\ 10^{-2}){}^{+3.0\%}_{-3.1\%}{}^{+6.6\%}_{-6.6\%}$
	**TW1**	$(3.105\ 10^{-2}){}^{+11.5\%}_{-9.7\%}{}^{+5.0\%}_{-5.0\%}$	$(3.899\ 10^{-2}){}^{+3.5\%}_{-4.0\%}{}^{+4.7\%}_{-4.7\%}$
	**TW2**	$(3.725\ 10^{-2}){}^{+10.1\%}_{-8.7\%}{}^{+24.7\%}_{-24.7\%}$	$(4.653\ 10^{-2}){}^{+3.2\%}_{-3.6\%}{}^{+23.1\%}_{-23.1\%}$

**Table 6:** LO and NLO QCD inclusive cross sections for single *X* and single *Y* production at the LHC, running at a center-of-mass energy of $\sqrt{s}=13$ TeV. The results are shown together with the associated scale and PDF relative uncertainties in the context of several benchmark scenarios

$m_{\scriptscriptstyle Q}$ [GeV]	Scenario	$\sigma _\mathrm{LO}$ [pb]	$\sigma _\mathrm{NLO}$ [pb]
400	**XW1**	$(2.483\ 10^{0}){}^{+3.1\%}_{-3.1\%}{}^{+1.8\%}_{-1.8\%}$	$(2.501\ 10^{0}){}^{+0.8\%}_{-0.4\%}{}^{+1.8\%}_{-1.8\%}$
	**XW2**	$(3.102\ 10^{0}){}^{+0.0\%}_{-0.7\%}{}^{+1.3\%}_{-1.3\%}$	$(3.217\ 10^{0}){}^{+1.2\%}_{-0.6\%}{}^{+1.3\%}_{-1.3\%}$
	**YW1**	$(1.361\ 10^{0}){}^{+3.3\%}_{-3.2\%}{}^{+2.8\%}_{-2.8\%}$	$(1.452\ 10^{0}){}^{+1.2\%}_{-0.4\%}{}^{+2.8\%}_{-2.8\%}$
	**YW2**	$(4.114\ 10^{0}){}^{+1.1\%}_{-1.5\%}{}^{+4.7\%}_{-4.7\%}$	$(4.278\ 10^{0}){}^{+1.1\%}_{-0.3\%}{}^{+4.6\%}_{-4.6\%}$
800	**XW1**	$(7.639\ 10^{-1}){}^{+6.4\%}_{-5.7\%}{}^{+2.0\%}_{-2.0\%}$	$(8.226\ 10^{-1}){}^{+0.9\%}_{-1.1\%}{}^{+2.0\%}_{-2.0\%}$
	**XW2**	$(5.408\ 10^{-1}){}^{+3.3\%}_{-3.5\%}{}^{+2.0\%}_{-2.0\%}$	$(6.002\ 10^{-1}){}^{+1.3\%}_{-0.8\%}{}^{+1.8\%}_{-1.8\%}$
	**YW1**	$(3.289\ 10^{-1}){}^{+6.9\%}_{-6.1\%}{}^{+3.8\%}_{-3.8\%}$	$(3.593\ 10^{-1}){}^{+1.1\%}_{-1.4\%}{}^{+3.7\%}_{-3.7\%}$
	**YW2**	$(7.827\ 10^{-1}){}^{+5.0\%}_{-4.7\%}{}^{+7.7\%}_{-7.7\%}$	$(8.688\ 10^{-1}){}^{+1.2\%}_{-1.2\%}{}^{+7.2\%}_{-7.2\%}$
1200	**XW1**	$(3.202\ 10^{-1}){}^{+8.3\%}_{-7.2\%}{}^{+2.2\%}_{-2.2\%}$	$(3.617\ 10^{-1}){}^{+1.5\%}_{-2.1\%}{}^{+2.2\%}_{-2.2\%}$
	**XW2**	$(1.420\ 10^{-1}){}^{+5.7\%}_{-5.4\%}{}^{+3.3\%}_{-3.3\%}$	$(1.658\ 10^{-1}){}^{+1.8\%}_{-1.8\%}{}^{+3.0\%}_{-3.0\%}$
	**YW1**	$(1.108\ 10^{-1}){}^{+9.0\%}_{-7.8\%}{}^{+5.1\%}_{-5.1\%}$	$(1.282\ 10^{-1}){}^{+1.9\%}_{-2.4\%}{}^{+4.8\%}_{-4.8\%}$
	**YW2**	$(2.245\ 10^{-1}){}^{+7.3\%}_{-6.5\%}{}^{+12.3\%}_{-12.3\%}$	$(2.618\ 10^{-1}){}^{+1.9\%}_{-2.2\%}{}^{+11.5\%}_{-11.5\%}$
1600	**XW1**	$(1.503\ 10^{-1}){}^{+9.7\%}_{-8.3\%}{}^{+2.4\%}_{-2.4\%}$	$(1.780\ 10^{-1}){}^{+2.3\%}_{-2.8\%}{}^{+2.4\%}_{-2.4\%}$
	**XW2**	$(4.489\ 10^{-2}){}^{+7.4\%}_{-6.7\%}{}^{+5.1\%}_{-5.1\%}$	$(5.474\ 10^{-2}){}^{+2.5\%}_{-2.6\%}{}^{+4.7\%}_{-4.7\%}$
	**YW1**	$(4.303\ 10^{-2}){}^{+10.6\%}_{-9.0\%}{}^{+6.9\%}_{-6.9\%}$	$(5.228\ 10^{-2}){}^{+2.9\%}_{-3.4\%}{}^{+6.4\%}_{-6.4\%}$
	**YW2**	$(7.740\ 10^{-2}){}^{+8.9\%}_{-7.8\%}{}^{+18.8\%}_{-18.8\%}$	$(9.423\ 10^{-2}){}^{+2.7\%}_{-3.0\%}{}^{+17.5\%}_{-17.5\%}$
2000	**XW1**	$(7.495\ 10^{-2}){}^{+10.8\%}_{-9.2\%}{}^{+2.8\%}_{-2.8\%}$	$(9.215\ 10^{-2}){}^{+3.0\%}_{-3.5\%}{}^{+2.7\%}_{-2.7\%}$
	**XW2**	$(1.577\ 10^{-2}){}^{+8.8\%}_{-7.8\%}{}^{+7.2\%}_{-7.2\%}$	$(1.997\ 10^{-2}){}^{+3.1\%}_{-3.2\%}{}^{+6.8\%}_{-6.8\%}$
	**YW1**	$(1.795\ 10^{-2}){}^{+11.9\%}_{-9.9\%}{}^{+9.3\%}_{-9.3\%}$	$(2.351\ 10^{-2}){}^{+4.0\%}_{-4.3\%}{}^{+8.9\%}_{-8.9\%}$
	**YW2**	$(2.968\ 10^{-2}){}^{+10.3\%}_{-8.9\%}{}^{+27.9\%}_{-27.9\%}$	$(3.752\ 10^{-2}){}^{+3.5\%}_{-3.8\%}{}^{+25.8\%}_{-25.8\%}$

## References

[1] Antoniadis I (1990). A possible new dimension at a few TeV. Phys. Lett. B.

[2] Kaplan DB (1991). Flavor at SSC energies: a new mechanism for dynamically generated fermion masses. Nucl. Phys. B.

[3] Arkani-Hamed N, Cohen AG, Katz E, Nelson AE, Gregoire T, Wacker JG (2002). The minimal moose for a little Higgs. JHEP.

[4] Contino R, Da Rold L, Pomarol A (2007). Light custodians in natural composite Higgs models. Phys. Rev. D.

[5] Matsedonskyi O, Panico G, Wulzer A (2013). Light top partners for a light composite Higgs. JHEP.

[6] ATLAS Collaboration, G. Aad et al., Search for pair and single production of new heavy quarks that decay to a $$Z$$ boson and a third-generation quark in $$pp$$ collisions at $$\sqrt{s}=8$$ TeV with the ATLAS detector. JHEP **11**, 104 (2014). arXiv:1409.5500

[7] ATLAS Collaboration, G. Aad et al., Search for production of vector-like quark pairs and of four top quarks in the lepton-plus-jets final state in $$pp$$ collisions at $$\sqrt{s}=8$$ TeV with the ATLAS detector. JHEP **08**, 105 (2015). arXiv:1505.04306

[8] ATLAS Collaboration, G. Aad et al., Search for pair production of a new heavy quark that decays into a $$W$$ boson and a light quark in $$pp$$ collisions at $$\sqrt{s} = 8$$ TeV with the ATLAS detector. Phys. Rev. D **92**(11), 112007 (2015). arXiv:1509.04261

[9] ATLAS Collaboration, Search for new physics using events with $$b$$-jets and a pair of same charge leptons in 3.2 fb$$^{-1}$$ of $$pp$$ collisions at $$\sqrt{s}=13$$ TeV with the ATLAS detector. ATLAS-CONF-2016-032

[10] CMS Collaboration, Search for single production of a vector like T quark decaying to a Higgs boson and a leptonically decaying top quark. CMS-PAS-B2G-15-008

[11] CMS Collaboration, Search for top quark partners with charge 5/3 at $$\sqrt{s}=13$$ TeV. CMS-PAS-B2G-15-006

[12] CMS Collaboration, Search for pair production of vector-like T quarks in the lepton plus jets final state. CMS-PAS-B2G-16-002

[13] del Aguila F, Perez-Victoria M, Santiago J (2000). Observable contributions of new exotic quarks to quark mixing. JHEP.

[14] G. Cacciapaglia, A. Deandrea, N. Gaur, D. Harada, Y. Okada, L. Panizzi, Interplay of vector-like top partner multiplets in a realistic mixing set-up. JHEP **09**, 012 (2015). arXiv:1502.00370

[15] K. Ishiwata, Z. Ligeti, M.B. Wise, New vector-like fermions and flavor physics. JHEP **10**, 027 (2015). arXiv:1506.03484

[16] Atre A, Azuelos G, Carena M, Han T, Ozcan E, Santiago J, Unel G (2011). Model-independent searches for new quarks at the LHC. JHEP.

[17] Buchkremer M, Cacciapaglia G, Deandrea A, Panizzi L (2013). Model independent framework for searches of top partners. Nucl. Phys. B.

[18] Alwall J, Frederix R, Frixione S, Hirschi V, Maltoni F, Mattelaer O, Shao HS, Stelzer T, Torrielli P, Zaro M (2014). The automated computation of tree-level and next-to-leading order differential cross sections, and their matching to parton shower simulations. JHEP.

[19] Alloul A, Christensen ND, Degrande C, Duhr C, Fuks B (2014). FeynRules 2.0—a complete toolbox for tree-level phenomenology. Comput. Phys. Commun..

[20] Degrande C (2015). Automatic evaluation of UV and R2 terms for beyond the Standard Model Lagrangians: a proof-of-principle. Comput. Phys. Commun..

[21] Hahn T (2001). Generating Feynman diagrams and amplitudes with FeynArts 3. Comput. Phys. Commun..

[22] Degrande C, Duhr C, Fuks B, Grellscheid D, Mattelaer O, Reiter T (2012). UFO—the Universal FeynRules output. Comput. Phys. Commun..

[23] Hirschi V, Frederix R, Frixione S, Garzelli MV, Maltoni F, Pittau R (2011). Automation of one-loop QCD corrections. JHEP.

[24] Frixione S, Kunszt Z, Signer A (1996). Three jet cross-sections to next-to-leading order. Nucl. Phys. B.

[25] Frederix R, Frixione S, Maltoni F, Stelzer T (2009). Automation of next-to-leading order computations in QCD: the FKS subtraction. JHEP.

[26] Frixione S, Webber BR (2002). Matching NLO QCD computations and parton shower simulations. JHEP.

[27] Cacciapaglia G, Deandrea A, Panizzi L, Gaur N, Harada D, Okada Y (2012). Heavy vector-like top partners at the LHC and flavour constraints. JHEP.

[28] Passarino G, Veltman MJG (1979). One loop corrections for e+ e$$-$$ annihilation into mu+ mu$$-$$ in the Weinberg model. Nucl. Phys. B.

[29] Ossola G, Papadopoulos CG, Pittau R (2008). On the rational terms of the one-loop amplitudes. JHEP.

[30] Christensen ND, de Aquino P, Degrande C, Duhr C, Fuks B, Herquet M, Maltoni F, Schumann S (2011). A comprehensive approach to new physics simulations. Eur. Phys. J. C.

[31] A.K. Alok, S. Banerjee, D. Kumar, S.U. Sankar, D. London, New-physics signals of a model with a vector-singlet up-type quark. Phys. Rev. D **92**, 013002 (2015). arXiv:1504.00517

[32] A.K. Alok, S. Banerjee, D. Kumar, S. Uma Sankar, Flavor signatures of isosinglet vector-like down quark model. Nucl. Phys. B **906**, 321–341 (2016). arXiv:1402.1023

[33] SLD Electroweak Group, DELPHI, ALEPH, SLD, SLD Heavy Flavour Group, OPAL, LEP Electroweak Working Group, L3 Collaboration, S. Schael et al., Precision electroweak measurements on the $$Z$$ resonance. Phys. Rep. **427**, 257–454 (2006). arXiv:hep-ex/0509008

[34] Cacciapaglia G, Deandrea A, Harada D, Okada Y (2010). Bounds and decays of new heavy vector-like top partners. JHEP.

[35] Deandrea A (1997). Atomic parity violation in cesium and implications for new physics. Phys. Lett. B.

[36] NNPDF Collaboration, R.D. Ball et al., Parton distributions for the LHC Run II. JHEP **04**, 040 (2015). arXiv:1410.8849

[37] Buckley A, Ferrando J, Lloyd S, Nordström K, Page B, Rüfenacht M, Schönherr M, Watt G (2015). LHAPDF6: parton density access in the LHC precision era. Eur. Phys. J. C.

[38] Demartin F, Forte S, Mariani E, Rojo J, Vicini A (2010). The impact of PDF and alphas uncertainties on Higgs production in gluon fusion at hadron colliders. Phys. Rev. D.

[39] Aliev M, Lacker H, Langenfeld U, Moch S, Uwer P, Wiedermann M (2011). HATHOR: HAdronic Top and Heavy quarks crOss section calculatoR. Comput. Phys. Commun..

[40] Artoisenet P, Frederix R, Mattelaer O, Rietkerk R (2013). Automatic spin-entangled decays of heavy resonances in Monte Carlo simulations. JHEP.

[41] J. Alwall, C. Duhr, B. Fuks, O. Mattelaer, D.G. Ozturk et al., Computing decay rates for new physics theories with FeynRules and MadGraph5/aMC@NLO. arXiv:1402.1178

[42] Sjöstrand T, Ask S, Christiansen JR, Corke R, Desai N, Ilten P, Mrenna S, Prestel S, Rasmussen CO, Skands PZ (2015). An introduction to PYTHIA 8.2. Comput. Phys. Commun..

[43] Cacciari M, Salam GP, Soyez G (2008). The anti-k(t) jet clustering algorithm. JHEP.

[44] Cacciari M, Salam GP, Soyez G (2012). FastJet user manual. Eur. Phys. J. C.

[45] ATLAS Collaboration, G. Aad et al., Search for the production of single vector-like and excited quarks in the $$Wt$$ final state in $$pp$$ collisions at $$\sqrt{s}= 8$$ TeV with the ATLAS detector. JHEP **02**, 110 (2016). arXiv:1510.02664

[46] ATLAS Collaboration, G. Aad et al., Search for single production of vector-like quarks decaying into Wb in pp collisions at $$\sqrt{s} = 8$$ TeV with the ATLAS detector. Eur. Phys. J. C **76**(8), 442 (2016). arXiv:1602.05606 10.1140/epjc/s10052-016-4281-8 PMC5331864 28303082

[47] ATLAS Collaboration, G. Aad et al., Search for single production of a vector-like quark via a heavy gluon in the $$4b$$ final state with the ATLAS detector in $$pp$$ collisions at $$\sqrt{s} = 8$$ TeV. Phys. Lett. B **758**, 249–268 (2016). arXiv:1602.06034

[48] ATLAS Collaboration, Search for single production of vector-like quarks decaying into $$Wb$$ in $$pp$$ collisions at $$\sqrt{s} =13$$ TeV with the ATLAS detector. ATLAS-CONF-2016-072

